# Impaired erythroid maturation in murine embryos upon loss of the preeclampsia-associated serine protease prostasin Prostasin loss impairs erythropoiesis

**DOI:** 10.1371/journal.pone.0360958

**Published:** 2026-10-16

**Authors:** Sara Di Carlo, Adrian Salas-Bastos, Mariela Castelblanco Castelblanco, Muriel Auberson, Dongmei Wang, Marie Rumpler, Malaury Tournier, Olivier Staub, Lukas Sommer, Silvia Ferreira Lopes, Ana Jiménez Sànchez, Olaia Naveiras, Edith Hummler

**Affiliations:** 1 Department of Biomedical Sciences, University of Lausanne, Lausanne, Switzerland; 2 Institute of Anatomy, Stem Cell Biology, University of Zurich, Zurich, Switzerland; 3 Hematology Service, Departments of Clinical Oncology and Laboratory Medicine and Pathology, Lausanne University Hospital (CHUV) and University of Lausanne, Lausanne, Switzerland; 4 Department of Fundamental Oncology, University of Lausanne, Lausanne, Switzerland; Laboratoire de Biologie du Développement de Villefranche-sur-Mer, FRANCE

## Abstract

In humans, the membrane-bound serine protease prostasin encoded by *Prss8* is associated with preeclampsia, a gestational hypertension disorder affecting blood supply of the placenta. Deficiency of *Prss8* in mice resulted in embryonic lethality by embryonic day (E) 14.5 and impaired maturation of placental labyrinth. We found in *Prss8*^*-/-*^ embryos at E11.5 and E12.5 a reduced frequency of both circulating primitive and erythro-myeloid progenitor (EMP)-derived definitive erythroblasts in the yolk sac, placenta and fetal liver. Single-cell RNA sequencing analyses revealed a downregulation of fetal hemoglobin genes and an upregulation of ribosomal genes in primitive erythroid cells of *Prss8*^*-/-*^ embryos, indicating a defective erythroid maturation. We suggest that prostasin regulates primitive erythropoiesis in a cell-extrinsic manner, since its expression was absent in circulating erythroid cells. Indeed, the yolk sac vascular remodeling of *Prss8*^*-/-*^ embryos was impaired as evidenced by reduced vessel branching and junctions, likely affecting placenta vascularization and contributing to embryonic demise. Our findings unveil a novel role for prostasin during developmental erythropoiesis and vascular patterning and provide insight into how *Prss8* dysregulation may contribute to placental pathologies such as preeclampsia.

## Introduction

Proteases, and especially serine proteases, have a pivotal role in signaling processes and are thus fundamental to survival across all living organisms [1]. Serine proteases represent the largest family of proteolytic enzymes and control several physiological processes including epithelial development, digestion, blood coagulation, inflammation and immunity. Likewise, there is growing evidence of their contribution to the pathogenesis of inflammatory and neoplastic diseases [2,3]. Prostasin is a glycosyl-phosphatidylinositol membrane-anchored serine protease expressed in the epithelial compartment of most tissues, including prostate, lung, kidney, colon and skin, where it was shown to play a tissue-specific role [4–6]. Prostasin was the first among many membrane-anchored serine proteases to be described as an activator of the epithelial Na^+^ channel (ENaC) [7–9], thereby also named channel-activating protease 1 (CAP1). While ENaC was found to be proteolytically cleaved by prostasin in *in vitro* experiments [10–12], this has not yet been confirmed *in vivo* [13]. Namely, prostasin has been shown to elicit a variety of non-proteolytic functions *in vivo*, such as epidermal barrier function and skin inflammation [6,14,15]. Consequently, both prostasin overexpression and downregulation have been linked to the onset of several diseases, including hypertension and cancer [16–21]. More recently, prostasin was also found to be dysregulated in females in a sex-specific manner and associated to coronary microvascular dysfunction in patients with heart failure [22]. Interestingly, a single nucleotide polymorphism (SNP) of *Prss8* (rs12597511) in women has been linked to preeclampsia, a severe form of hypertension during pregnancy, leading to impaired placentation and premature embryonic death [23,24]. Previous studies described a role of prostasin in human trophoblast differentiation, although its implication in embryonic development has not yet been extensively investigated [25–28].

During embryonic development in mammals, erythropoiesis results in the rapid production of erythroid cells that support the growth and survival of the embryo and fetus [29]. Three waves of erythropoiesis have been described to occur in the murine embryo. The first one, prior to the onset of embryonic circulation at E7.5 and to the establishment of the hematopoietic system, is called “primitive” and takes place in the yolk sac giving rise to mature red blood cells (RBCs), megakaryocytes and macrophages [30,31]. Concurrently, the second wave of multipotent hematopoietic progenitor cells, termed “erythro-myeloid progenitors” (EMP) [32,33] emerges in the yolk sac and serves as major source of HSC-independent hematopoiesis in the developing embryo [34]. *Bona fide* hematopoietic stem cells (HSCs) capable of producing all lympho-myeloid lineages arise only at late embryonic/early fetal stage of development (E10.5 - E11.5) and derive from hemogenic endothelium in the major intra- and extraembryonic arteries including the AGM and placenta. This third wave supplies adult-type erythrocytes and is thus referred to as “definitive” erythropoiesis [29]. While primitive erythrocytes enter circulation in their nucleated form when the heartbeat starts and enucleate in the bloodstream between E12.5 and E16.5, both EMP-derived and HSC-derived definitive erythrocytes circulate exclusively as enucleated cells and mature extravascularly in the fetal liver or subsequently in the bone marrow [35]. However, the complex nature of these processes has only been elucidated in the past decades, and the precise origin of the different erythropoietic waves remains still debated [36–38]. In this study, we aimed to explore the physiological role of prostasin in murine embryos prior to their premature death by comparing the phenotype and transcriptome of *Prss8*^*+/+*^ (WT) to *Prss8*^*-/-*^ (KO) embryos. Due to the previously observed defect in vascularization and suspected impaired blood supply of the placenta, we focused on the main hematopoietic tissues including yolk sac, AGM, fetal liver and placenta. Our data demonstrated that lack of prostasin led to a reduction of primitive erythroblasts and significant transcriptomic variation in these cells. The downregulation of fetal hemoglobin genes at E11.5 in the embryo proper was associated with aberrant terminal maturation of primitive erythroid cells. Overall, this resulted in the impaired vessel remodeling of the yolk sac at E12.5 and embryonic lethality. Finally, our findings unwrap new research perspectives on the physiological role of prostasin as well as on the regulation of embryonic erythropoiesis.

## Methods

### Mice

The generation of mice carrying one knockout allele of *Prss8* has been described previously [39]. *Prss8*^*-/-*^ and *Prss8*^*+/+*^ embryos were generated by intercrossing *Prss8*^***+/-***^ mice. Mating was started in the evening, and females were checked for vaginal plugs the next morning, with the morning of plug defined as E0.5. Pregnant mice were sacrificed at E9.5, E10.5, E11.5 and E12.5 and euthanasia was achieved by pentobarbital injection (adults) or decapitation (embryos). Embryos were collected for the analysis of yolk sac, AGM, fetal liver or placenta. The viability of embryos was assessed by heart beating and/or gross morphology. Genotyping of *Prss8* was performed by PCR using the following primers for *Prss8* (sense 1: 5’- GCAGTTGTAAGCTGTCATGTG-3’; antisense: 5’- TCCAGGAAGCATAGGTAGAAG-3’, sense 2: 5’- CAGCAGCTCGAGGTACCACTC −3’). All animal experiments in this study were conducted according to Swiss guidelines with approval of the cantonal authorities (Service de la consommation et des affaires vétérinaires, Etat de Vaud, authorization numbers VD3812a-c). The mice were kept in an approved animal care facility of the University of Lausanne with free access to food (standard diet, SD, 0.25% Na+ and 0.70% K+) and tap water.

### Single cell suspension

Cells from yolk sac, AGM or fetal liver were isolated by placing each organ in one 24-well with 300 µL of dissociation medium (1 mg/ml dispase-collagenase in PBS + 5 mM Ca^2+^) at 37°C for 40–45 minutes. The tissues were gently crushed every 10 min using a 1000 µL pipette until complete tissue disruption. The cell suspension was collected and passed through a 40 µm cell strainer (Greiner Bio-One). The cell suspension was centrifuged at 1200 rpm for 5 minutes and resuspended in PBS + 10% FCS.

### FACS analysis

The single cell suspension was stained with following antibodies: FITC Ter-119 (1:500; 557915, BD Pharmingen); PE/Cy7 CD71 ab (1:200; 113812, BioLegend); FITC CD41 ab (1:200; 561849, BD Pharmingen); PE/Cy7 CD43 ab (1:100; 121218, BioLegend); BUV737 CD45.2 ab (1:100; 1313859, BD Horizon); PE CD31 ab (1:200; 12-0311-82, Invitrogen); A700 c-kit ab (1:200; 56-1172-82, Invitrogen); live/dead aqua staining (1:1000; L34957 Invitrogen). Other antibodies used for macrophages, neutrophils, NK cells, T- and B- cells were the following: BV650 CD3e (1:500; AB_2738779, BD Horizon); FITC NK-1 (1:200; AB_394676, BD Pharmingen); PerCP/Cy5.5 CD11c (1:200; 117328, BioLegend); BV421 CD11b (1:100; 101251, BioLegend); PE-texas red F4/80 (1:500; AB_10372055, Invitrogen); APC/Cy7 Ly6G (1:500; 560600, BD Pharmingen); BV711 B220 (1:200; AB_2738470, BD Horizon). Fluorescent signal was recorded from BD LSRFortessa with the BD FACSDiva software. Single color controls for each fluorochrome were acquired to generate the compensation matrix that was applied to all the experimental files prior to analysis with FlowJo software (Version 10.8.1). Gating strategy for pre-HSCs and hemogenic endothelium was performed as in [40].

### Imaging flow cytometry

Samples were acquired on a 2 camera, 12 channel ImageStreamX imaging flow cytometer (Cytek Biosciences) at low speed and highest magnification (60X). Cells were excited using a 405 nm laser (25mW), a 488 nm laser (150mW), a 561 nm laser (150mW), a 642 nm laser (120mW) and a 785nm Side Scatter (SSC) laser (3 mW). Only events with a bright field area greater than 1 µm² (to exclude cell debris) and non-saturating pixels (Raw max pixel values below 4096) were collected. Data was acquired for at least 10000 events/sample. Experimental samples contained images for bright field (Channels 1 and 9), Ter119-FITC (Channel 2), CD71-PECy7 (Channel 6), Hoechst 33342 (Channel 7), and Side Scatter (Channel 12). The antibodies used are the same as listed in the section “FACS analysis”. Single color controls for each fluorochrome were acquired to generate the compensation matrix that was applied to all the experimental files prior to analysis using IDEAS (Image Data Exploration and Analysis Software) 6.2 software (Cytek Biosciences). For the data analysis of placenta and yolk sac, we first selected erythrocyte populations based on Ter119 and Hoechst. Then, Ter119^+^Hoechst^+^ cells were selected based on CD71 higher intensity to 10^4^. Moreover, cell images were analyzed to confirm cell morphology and staining diffusion. Dead cells had high diffusion of Ter119 and CD71 antibodies, suggestive of membrane permeability. For the data analysis of fetal liver samples, we referred to a previous publication [41].

### Histology

Following dissection, yolk sac and placenta were fixed overnight in 4% PFA and embedded in paraffin. 4 µm paraffin sections were stained with H&E to assess the general morphology. The staining was performed on the Sakura Tissue-Tek Prisma® Plus Automated Slide Stainer linked to the Tissue-Tek® Glas™ g2 Automated Glass Cover slipper. Images were acquired using the Zeiss Axioscan 7 microscope with a resolution of 40X. For the collection of circulating erythroid cells, vitelline and umbilical arteries were cut in 300 µL PBS + 0.01% Heparin in a 48 well plate. Then embryonic blood cells were collected from each well for Giemsa staining and visualized using the HSP-1000 high resolution microscope.

The yolk sac was placed in 4% PFA overnight. After 24 hours, the tissue was washed 3x with PBS and blocked at RT in staining buffer (PBS + 0.3% Triton-X + 3% BSA) for 1h. It was then stained with anti-mouse CD31 (1:500; 553370, BD Bioscience), anti-rat Ter119 (1:200; 14-5921-82, Invitrogen) and anti-mouse VE-Cadherin (1:200; AF1002, R&D systems) antibodies overnight, followed by incubation with secondary antibodies for 1h at RT. Finally, the yolk sac was placed in mounting medium containing 4% DAPI on a glass slide and flat opened for microscopic analysis. Whole mount images were taken using a Nikon Ti2 spinning disk microscope with a resolution of 40X.

### Western blotting

Embryonic tissues were collected into 2 ml Eppendorf tubes, flash frozen in liquid nitrogen, and stored at −80°C. For protein extraction, we used the following extraction buffer: TrisHCl pH7.5, 50mM; EDTA 1mM; EGTA 1mM; sucrose 0.27M plus protease inhibitors (complete, Roche # 11836145001) and phosphatase inhibitors (phosSTOP, Sigma # 04 906 837 001). 100 µl was added directly to the tubes containing the tissues. Tissues were then homogenized with a bead mixer for 1.5 min and incubated for 30 min on ice. Protein homogenates were centrifuged at 13000 rpm for 15 minutes at 4°C, supernatants were then transferred in new tubes and protein concentration was measured using the Bradford assay. For WB, 15 µg of protein was used for each condition, boiled for 10 min at 95°C before loading on 4–15% Criterion TGX Precast Midi Protein Gel (Bio-Rad 5671085). Proteins were separated for 2h at 100V and then transferred to nitrocellulose membranes for 2.5h at 100V using a wet transfer apparatus (Bio-Rad). Membranes were blocked for 1h in 2% milk, prepared with PBS, and incubated with the following primary antibodies overnight at 4°C: Anti-prss8 (15527–1-AP, proteintech), Anti-β-Actin (1:500, mouse monoclonal, A1978-200UL, sigma), Anti-Tubulin (1:10000, mouse monoclonal, T5201-200UL, sigma). Secondary antibodies were applied for 1h at room temperature: Peroxidase-conjugated AffiniPure Goat Anti-Rabbit IgG (H + L) (111-035-144, Jackson Immuno Research), 1:5000 Peroxidase-conjugated AffiniPure Goat Anti-Mouse IgG (H + L) (115-035-003, Jackson Immuno Research), 1:5000. WesternBright Quantum – HRP Substrate - 200mL, K-12042-D20, Witec.

### Colony Forming Unit (CFU) assay

Single cell suspension was obtained as described previously and cells were counted and diluted to a final concentration of 5 x 10^5^ cells/mL. Then, 250 µL of cell suspension was added to 2.5 mL of MethoCult GF M3434 (3434, Stem Cell Technologies) or SF M3436 medium (3436, Stem Cell Technologies) and 1.1 mL/well was plated in duplicate in a SmartDish™ 6-well culture plate (27370, Stem Cell Technologies). Water was added into empty wells to minimize the medium evaporation. The read out was performed after 8 days of culture with the StemVision automated microscope (Stem Cell Technologies). Each dot represents the average of the two technical replicates.

For the fetal liver derived cells, an extra plate was seeded with a third of the whole tissue single cell suspension to determine total number of CFUs per FL cells. After 7 days of culture in M3434, the full content of each well was recovered and methylcellulose removed by 5x washing with PBS + 10% FCS. Cells were then centrifuges at 1200 rpm for 5 minute and stained for FACS analysis as described using the following antibodies: FIT CD71 (1:200; 561849, BD Pharmingen), APC Gr1 (1:200; 304102, Biolegend) and APC F4/80 (1:200; 304099, Biolegend), AF700 Ter119 (1:500; 304128, Biolegend), BV412 CD41 (1:200; 133911, Biolegend), BUV395 CD45 (1:200; 564279, BD).

#### Quantitative reverse transcription PCR (qRT-PCR).

Tissues were flash frozen at −80°C after dissection. For RNA extraction, the tissue was disrupted using metallic balls and TissueLyser at 30 mut/sec for 15 seconds and RNA was isolated using the RNeasy Midi Kit (75144, Qiagen). RNA quality was assessed using Nanodrop spectrometry and 1 µg of RNA was converted into cDNA using the PrimeScript RT reagent kit (Perfect Real Time) (RR037A, Takara) according to manufacturer’s instructions. Gene expression was determined using SYBER Green detection system and the following primers, *Prss8*: For, 5’- TGACCATTCTGCTCCTTCTC-3’, Rev, 5’- GACACCACCCATTTATTTGAC-3’; *Gapdh*: For, 5’- CCACCCAGAAGACTGTGGAT-3’, Rev, 5’- CACATTGGGGGTAGGAACAC-3’. Controls using RNA-free water were negative for targets and housekeeping genes. Fold change was calculated using ΔCt method with Gapdh as an internal standard and then calculating the ΔΔCt comparing the expression in the AGM to the yolk sac.

### Bulk RNA-sequencing

RNA was extracted as for qRT-PCR and RNA quality was assessed on a Fragment Analyzer (Agilent Technologies). The RNAs had RQNs between 8.7 and 10. RNA-seq libraries were prepared from 500 ng of total RNA with the Illumina TruSeq Stranded mRNA reagents (Illumina). Library preparation was performed on a Sciclone liquid handling robot (Revvity) using the provided automated script. Unique dual indexes were used for barcoding of the libraries. Libraries were quantified by a fluorometric method (QubIT, Life Technologies) and their quality assessed on a Fragment Analyzer (Agilent Technologies). Sequencing was performed on an Illumina NovaSeq 6000. Sequencing data were demultiplexed using the bcl2fastq2 Conversion Software (version 2.20, Illumina).

### Single-cell (sc) RNA-sequencing sample processing

A Chromium Next GEM Chip G (10X genomics) was loaded with the appropriate number of cells, and the sequencing libraries prepared with the Chromium Single Cell 3’reagent kit v3.1 dual index, strictly following the manufacturer’s recommendations. Briefly, an emulsion encapsulating single cells, reverse transcription reagents and cell barcoding oligonucleotides was generated. After the actual reverse transcription step, the emulsion is broken and double stranded cDNA generated and amplified in a bulk reaction (12 PCR cycles). This cDNA was fragmented, a P7 sequencing adaptor ligated, and a 3’ gene expression library generated by PCR amplification (13 cycles). Libraries were quantified by a fluorometric method, and their quality assessed on a Fragment Analyzer (Agilent Technologies). Sequencing was performed on Illumina NovaSeq 6000 v1.5 flow cells for 28-10-10-90 cycles (read1 – index i7 - index i5 - read2) with 1% PhiX spike in.

### Statistical analysis

The data shown are mean ± SEM of at least three biological replicates. Comparison between two or more groups was performed by two-tailed *t* test and values with *P* < 0.05 were considered statistically significant. All representations and statistical analyses were done using GraphPad Prism (version 9.3.1). Bulk RNA sequencing experiment was performed using two independent biological replicates, while single-cell RNA sequencing was performed once for each genotype and time point by pooling 3–4 embryos (for detailed methods, see supplemental data). Images were selected from matched embryos within a single experimental replicate to be representative of the most abundant expression pattern for each condition.

**Bulk** and **single-cell RNA sequencing** data **analyses and bioinformatics** were performed as described in the supplemental data.

## Results

### Vessel remodeling is impaired in the yolk sac of *Prss8*^*-/-*^ embryos

Previous results reported embryonic lethality of *Prss8-*deficient embryos by E14.5 [24]. While in the adult, prostasin is mainly expressed in epithelia [42], its expression during embryonic development has not yet been investigated. We observed that in embryos *Prss8* expression was significantly higher in WT yolk sac as compared to the AGM, and maximal at E10.5 (Fig 1A). Congruently, at the protein level prostasin was found to be expressed uniquely in the yolk sac of WT embryos (Fig 1B), coinciding with the site of primitive and EMP-definitive erythropoiesis. Prostasin was present in the yolk sac between E9.5 and E12.5 (Figs 1C and D), while absent in circulating erythroid cells (Fig 1E). Furthermore, as revealed by sc-RNA seq analysis of YS at E11.5 (S1A and S1B Figs) *Prss8* gene expression was not detected either in erythroid cells at the RNA level, but found exclusively within the extraembryonic endoderm (EXE) cluster. Notably, endothelial cells equally lacked *Prss8* expression, despite their known role in supporting developmental erythropoiesis (S1B Fig). While ENaC expression was lacking (notably *Scnn1g*), *F2rl1* (encoding for protease-activated receptor 2, PAR2) was found to be expressed by the same EXE cell cluster as *Prss8* (S1B Fig), indicating a possible role downstream of prostasin. The gross morphology of yolk sacs from E12.5 KO embryos revealed a pale vasculature, without an evident vascular network (Fig 2A). Histological examination and CD31 immune staining of whole mount yolk sacs of KO embryos exhibited an impaired vascular network at E12.5 (Figs 2B and E; Fig 3A). Vascular quantification at E12.5 revealed a significantly reduced number of vessel branches and junctions (Fig 2C). While these vascular defects were evident at E12.5, they were less pronounced at the earlier developmental stages E10.5 and E11.5 (Figs. 2D,E), suggesting that initial vasculogenesis is largely preserved in the absence of prostasin. Nevertheless, subtle vascular alterations may already be present at E11.5. Moreover, the frequency of endothelial cells (CD45^-^CD43^-^; CD31^+^Ve-cad^+^) detected in the yolk sac was comparable between the two genotypes (Fig 2F), confirming that blood vessel formation may be intact. Next, to analyze the transcriptome of the yolk sac during this developmental period we performed bulk RNA sequencing (RNA-seq) at E10.5 and E11.5. Differentially expressed gene (DEG) analysis comparing the two time points for each genotype identified a set of genes significantly and uniquely downregulated at E11.5 in the yolk sac of KO embryos (Fig 2G). Some of the downregulated genes in the KO were indeed associated with biological processes such as angiogenesis and vasculogenesis (Fig 2H), while such differences were not prevalent in the WT (Fig 2I). Genes represented in the GO term vasculogenesis and angiogenesis included transcription factors and regulatory proteins, such as *Tie1*, *Tek*, *Angpt1* and *Nos3,* known as primary biological mediators of vascular remodeling (S2A-D Figs). Thus, we concluded that embryonic lethality of *Prss8-*deficient embryos was likely not associated with defective vasculogenesis, but rather with lack of proper vessel remodeling.

**Fig 1:**
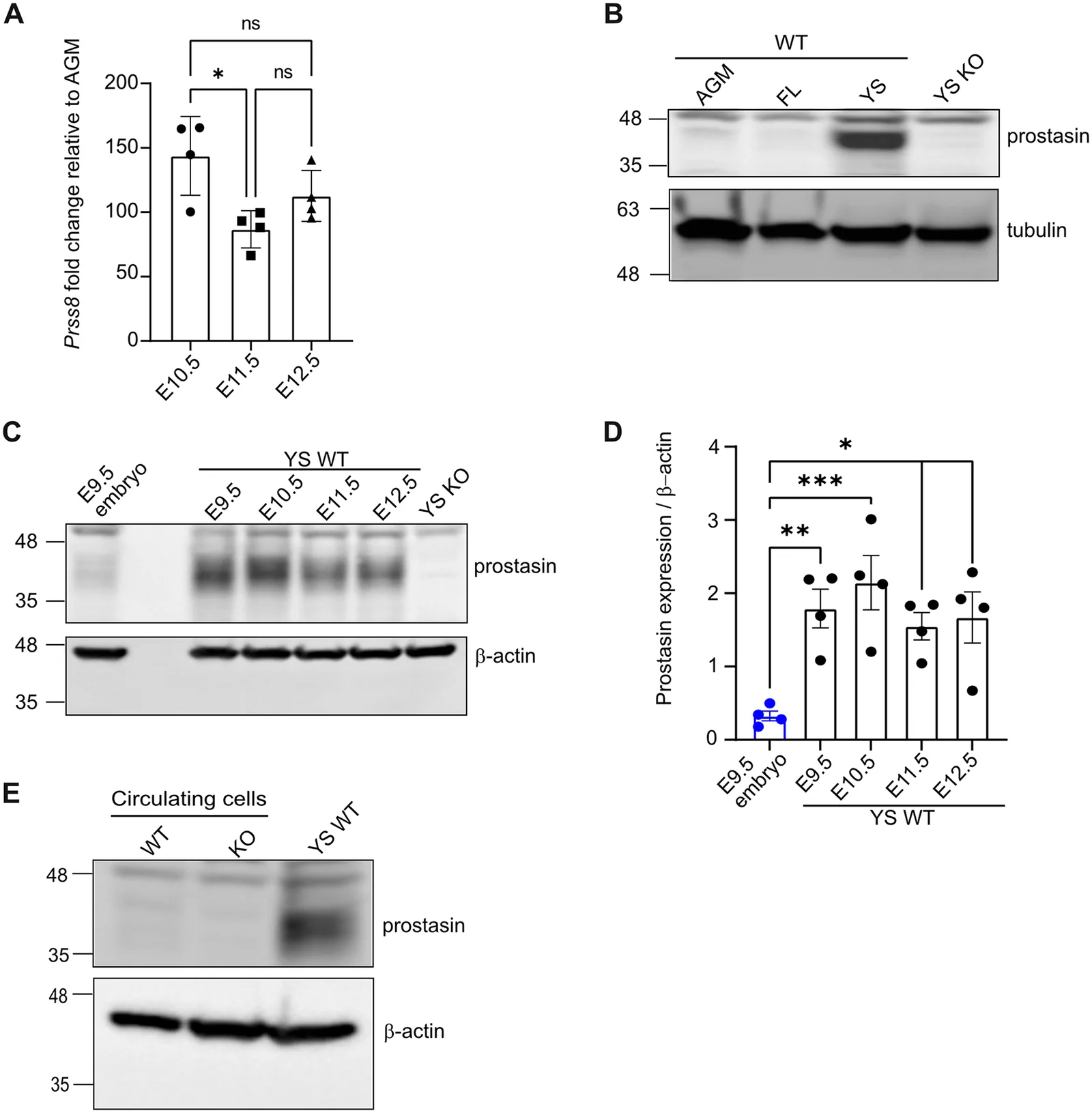
Prostasin expression during embryonic development. **(A)** qRT-PCR analysis of *Prss8* expression in WT yolk sac relative to the AGM at E10.5, E11.5 and E12.5. Shown is the fold change calculated as 2^(-ΔΔCT); n = 4. **(B)** Western blot analysis of prostasin expression in yolk sac, AGM and fetal liver from E11.5 embryos. **(C)** Western blot analysis and **(D)** quantification of prostasin expression in E9.5 whole WT embryo and yolk sac (WT; E9.5-E12.5); n = 4. **(E)** Prostasin expression in circulating blood cells at E11.5 analyzed by Western blot. n: biological replicates (every dot represents one biological replicate), values in bar plots are presented as the mean ± SEM. Significance was calculated using the unpaired Student t test and all graphs were plotted using GraphPad Prism 8.3.1. *P ≤ .05, **P ≤ .01, ***P ≤ .001. The images are representative of three biological replicates.

**Fig 2:**
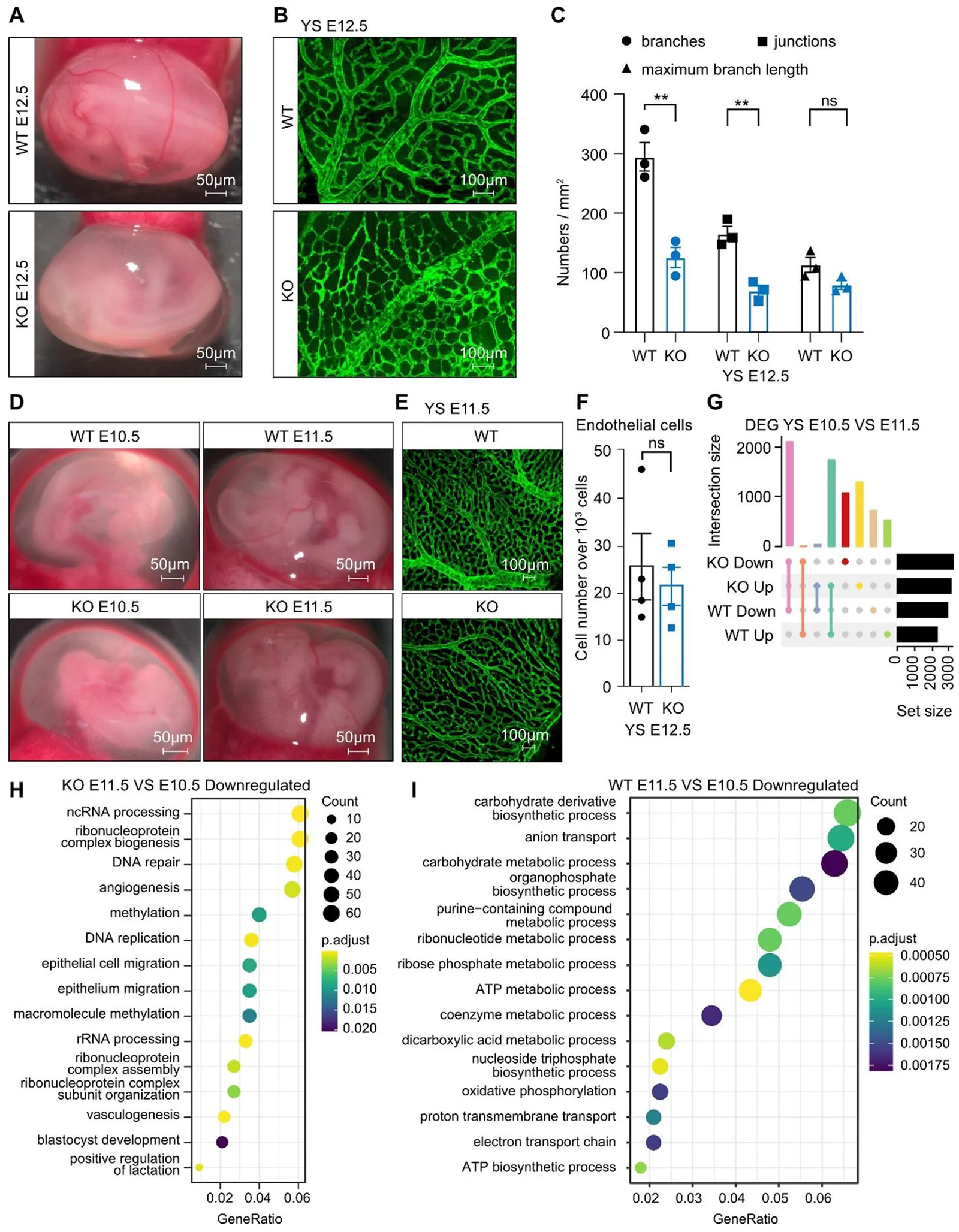
Changes in vasculogenesis-related genes correlated with impairment of vessel remodeling in the yolk sac of *Prss8*^-/-^ embryos. **(A, D)** Gross morphology of **(A)** E12.5 and **(D)** E10.5 (left) and E11.5 (right) *Prss8*^*+/+*^ (WT) and *Prss8*^*-/-*^ (KO) embryos; note that fully formed branching vessels are only visible in the WT embryo; scale bar: 50 µm. **(B, E)** CD31(GFP) immunostaining of whole mount yolk sac (YS) from **(B)** E12.5 and **(E)** E11.5 WT and KO embryos; scale bar: 100 µm. Magnification 40X and z-stack using a Nikon Ti2 and merged using Imaris software. **(C)** Vascular quantification of vessel network at E12.5; analysis performed using AnalyzeSkeloton in ImageJ; n = 3 **(F)** Number of endothelial cells (CD31^+^VEcad^+^) from yolk sac of E11.5 WT and KO embryos gated on CD45^-^CD43^-^ and quantified as cell number over 10^3^ cells by flow cytometry; n = 3 Fluorescent signal was recorded with the DIVA acquisition software using FACS Fortessa machine. **(G)** Upset plot of shared and differentially down- or upregulated genes at E11.5 compared to E10.5 yolk sac from KO and WT embryos (RNA-seq data; padj < 0.05). Vertical bars show the number of shared and unique genes in each comparison. **(H, I)** Dot plot showing the enrichment analysis (GO biological processes) of uniquely downregulated genes in the yolk sac at E11.5 compared to E10.5 from **(H)** KO and **(I)** WT embryos. n: biological replicates (every dot represents one biological replicate); values in bar plots are presented as the mean ± SEM. Significance was calculated using the unpaired Student t test and all graphs were plotted using GraphPad Prism 8.3.1. **P ≤ .01. The images are representative of three biological replicates.

**Fig 3:**
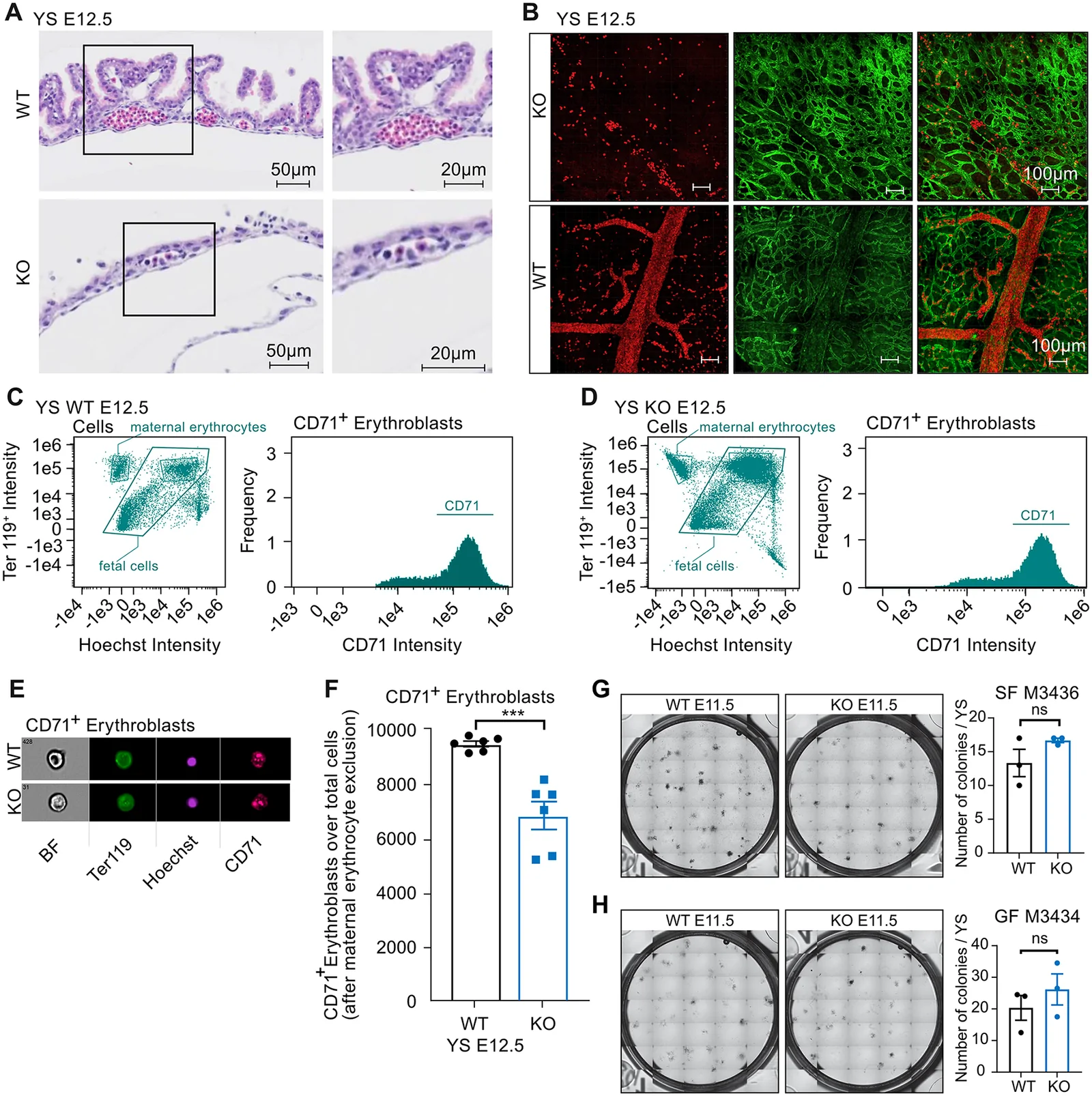
Fewer erythroblasts were present in the yolk sac from E12.5 *Prss8*^-/-^ embryos. Representative images of **(A)** H&E-stained sections of yolk sac (YS) from WT and KO embryos; scale bars: 50 µm and 20 µm, respectively. Magnification 40X, zoom using Zen Blue software, z-stack using Zeiss Axioscan 7. **(B)** Immunostaining of whole mount E12.5 yolk sac from WT and KO using antibodies against Ter119 (in red) and VE-Cadherin (in green); scale bar: 100 µm. Magnification 40X and z-stack using a Nikon Ti2, merged using Imaris software. **(C-F)** Imaging flow cytometry results for yolk sac-derived cells isolated from E12.5 **(C)** WT and **(D)** KO embryos. Representative dot plot histograms of Ter119, CD71 and Hoechst-stained cells are shown. **(E)** Representative images of CD71^+^ Hoechst^+^ erythroblasts from WT and KO embryos, respectively. Samples were acquired on ImageStreamX imaging flow cytometer (Cytek Biosciences) and analyzed using DIVA acquisition software. **(F)** Quantification of CD71^+^ Hoechst^+^ erythroblasts as cell number over total cells after maternal erythrocyte exclusion; n = 6. **(G, H)** Colony formation unit (CFU) assays and their quantifications from E11.5 WT and KO embryos using **(G)** selective erythroid progenitor (MethoCult, M3436) or **(H)** full hematopoietic progenitor medium (MethoCult, M3434); n = 3. n: biological replicates (every dot represents one biological replicate). Values in bar plots are presented as the mean ± SEM. Significance was calculated using the unpaired Student t test and all graphs were plotted using GraphPad Prism 8.3.1. ***P ≤ .001. The images are representative of three biological replicates.

### Reduction of primitive erythroblasts in *Prss8*^*-/-*^ embryos

The vasculature continuously undergoes dynamic structural changes (remodeling) to ensure stable vascular adaptation, which can be influenced by blood flow [43,44]. H&E staining of the yolk sac at E12.5 revealed the presence of nucleated erythroblasts within vessels of WT embryos, while they were rarely detected in the KO (Fig 3A). At E12.5, VE-cad/Ter119 immunostaining of yolk sac erythrocytes revealed a decrease in circulating erythroid cells in KO embryos (Fig 3B). This was further analyzed by imaging flow cytometry, using markers for Ter119, CD71 and Hoechst (Fig 3C and D). Nucleated erythroblasts (Ter119^+^CD71^+^Hoechst^+^) exhibited a regular shape and consistent Hoechst nuclear signal in both genotypes, indicative of functional cells (Fig 3E). We found that the number of erythroblasts (Ter119^+^CD71^+^Hoechst^+^) was significantly lower in yolk sacs of KO embryos (Fig 3F). Despite the reduced number of erythroblasts, E11.5 yolk sac-derived cells retained their colony forming capacity *in vitro* (Fig 3G and H). Notably as compared to the WT, expression of fetal globin genes did not decline for any erythroid cluster in the KO YS (S1C and D Fig). Since the embryonic circulation has been established by E12.5, the erythrocyte content was also analyzed in placentas at the same timepoint. H&E staining of placenta labyrinth from WT and KO embryos exhibited the presence of nucleated and enucleated erythrocytes (Fig 4A). Adult erythropoiesis occurs primarily in the bone marrow leading to full enucleation, while nucleated cells are uniquely derived from embryonic circulation, the majority of which consist of primitive erythroblasts at E12.5 [45]. Quantification of the number and area covered by nucleated erythroblasts showed that significantly fewer functional cells were present in KO embryos (Fig 4B). Thus, the overall number of embryonic primitive erythroblasts is lower in absence of prostasin, suggesting that this difference may contribute to the defective placenta development [24]. Imaging flow cytometry of E12.5 placentas revealed no effect on the cellular morphology of erythroblasts (Ter119^+^CD71^+^Hoechst^+^) in both genotypes (Figs 4C-E), even though their number was significantly reduced in KO embryos (Fig 4F). Taken together, these results indicate that, beyond previously described defects in placental vasculogenesis, lack of prostasin affects the amount of circulating primitive erythroblasts at E12.5.

**Fig 4:**
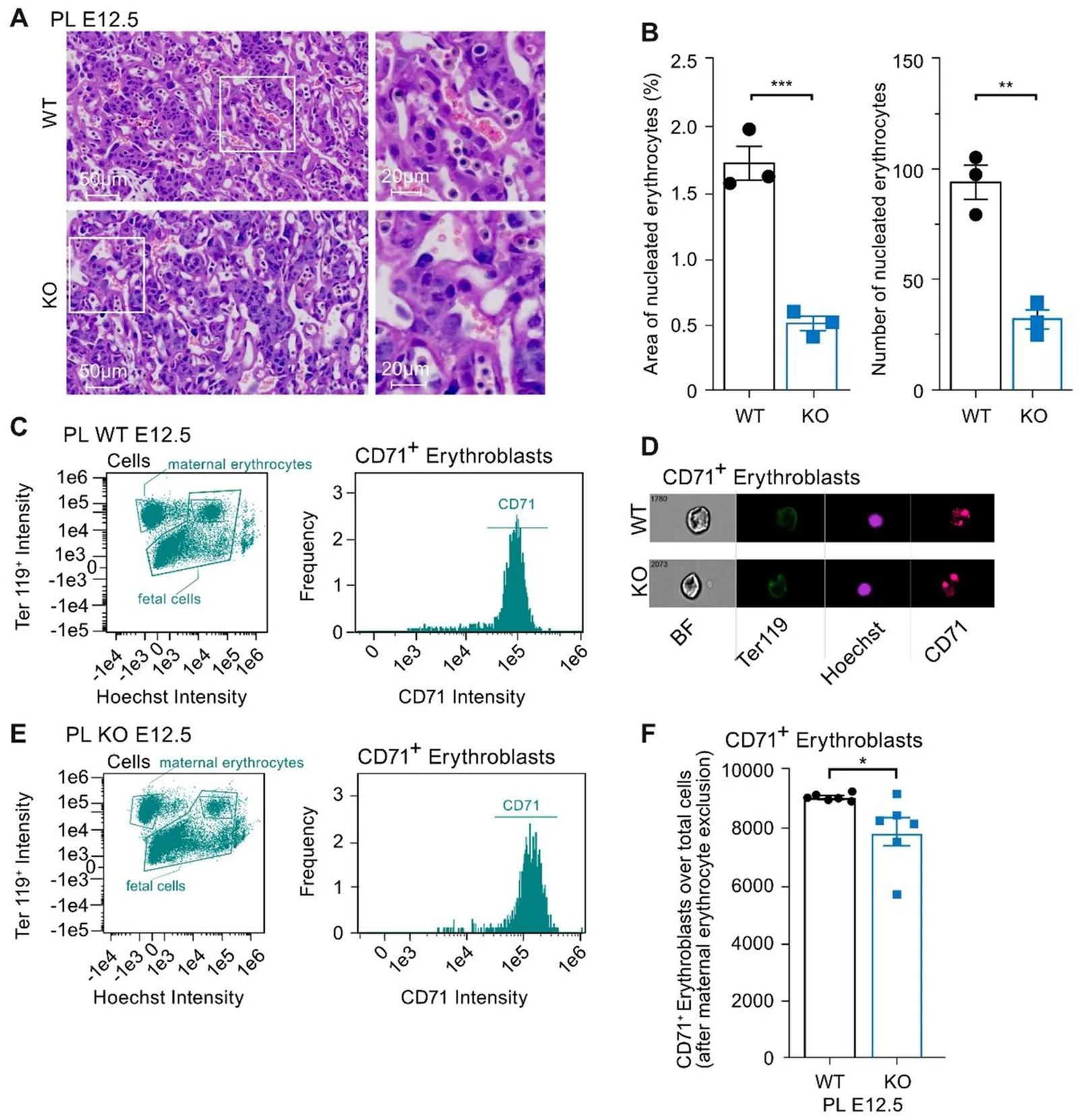
*Prss8*^-/-^ embryos exhibited fewer fetal erythrocytes within E12.5 placenta. **(A)** Representative H&E-stained images and their magnification of placental labyrinths (PL) from E12.5 WT and KO embryos; note areas of enucleated erythrocytes (light pink, maternal vasculature) are distinct from areas of nucleated erythroblasts (dark violet, fetal vasculature) are observed. Scale bars: 50µm and 20µm, respectively. Magnification 40X, zoom using Zen Blue software and z-stack using Zeiss Axioscan 7. **(B)** Quantification of nucleated erythroblast areas in placenta from WT and KO embryos using ImageJ; n = 3 **(C-F)** Imaging flow cytometry results for placenta-derived cells isolated from E12.5 **(C)** WT and **(E)** KO embryos. Representative dot plot histograms of Ter119 (FITC), CD71 (Pe-Cy7) and Hoechst-stained cells are shown. **(D)** Representative images of CD71^+^ Hoechst^+^ erythroblasts from WT and KO embryos, respectively. Samples were acquired on ImageStreamX imaging flow cytometer (Cytek Biosciences) and analyzed using DIVA acquisition software. **(F)** Quantification of CD71^+^ Hoechst^+^ erythroblasts as cell number over total cells after maternal erythrocyte exclusion; *n* = 6. n: biological replicates (every dot represents one biological replicate). Values in bar plots are presented as the mean ± SEM. Significance was calculated using the unpaired Student t test and all graphs were plotted using GraphPad Prism 8.3.1. *P ≤ .05, **P ≤ .01, ***P ≤ .001. The images are representative of three biological replicates.

### Primitive erythroid cells show defective maturation and impaired fetal hemoglobin expression in absence of prostasin

Primitive erythropoiesis occurs within the bloodstream, progressing from basophilic to polychromatic to orthochromatic erythroblasts. In both WT and KO embryos we found a majority of polychromatic and orthochromatic erythroblasts, with only a small percentage of basophilic erythroblasts (Fig 5), suggesting a distinct effect of prostasin loss in maturing erythroblasts versus hematopoietic progenitors. Cells from KO embryos showed the presence of cytoplasmic vacuoles especially in basophil and polychromatic progenitors (Fig 5A and B), which were very rare in WT counterparts. This finding suggested intrinsic damage and dysfunction of primitive erythroblasts in the KO. Next, to uncover transcriptional shifts that may support the erythropoiesis defect in KO embryos, we performed RNA-seq of AGM at E10.5 and E11.5. DEG analysis of data comparing E10.5 and E11.5 embryos identified a set of genes significantly and uniquely downregulated in the AGM of KO embryos at E11.5 compared to the WT (Fig 5C). Most of the downregulated genes were associated with microtubule organization and protein translation (Fig 5D), two crucial processes required for cellular remodeling during late stages of terminal erythropoiesis [46]. Further analysis of RNA-seq data indicated a significant upregulation of genes implicated in “response to hypoxia” at E11.5 in both AGM and yolk sac of KO embryos, such as *Hif1a*, *Cited2*, *Ddit4* and *Pdk1* (S3A and B Figs). Overall, these data showed that the hypoxic phenotype of *Prss8*^*-/-*^ embryos was associated with the defective terminal erythropoiesis.

**Fig 5:**
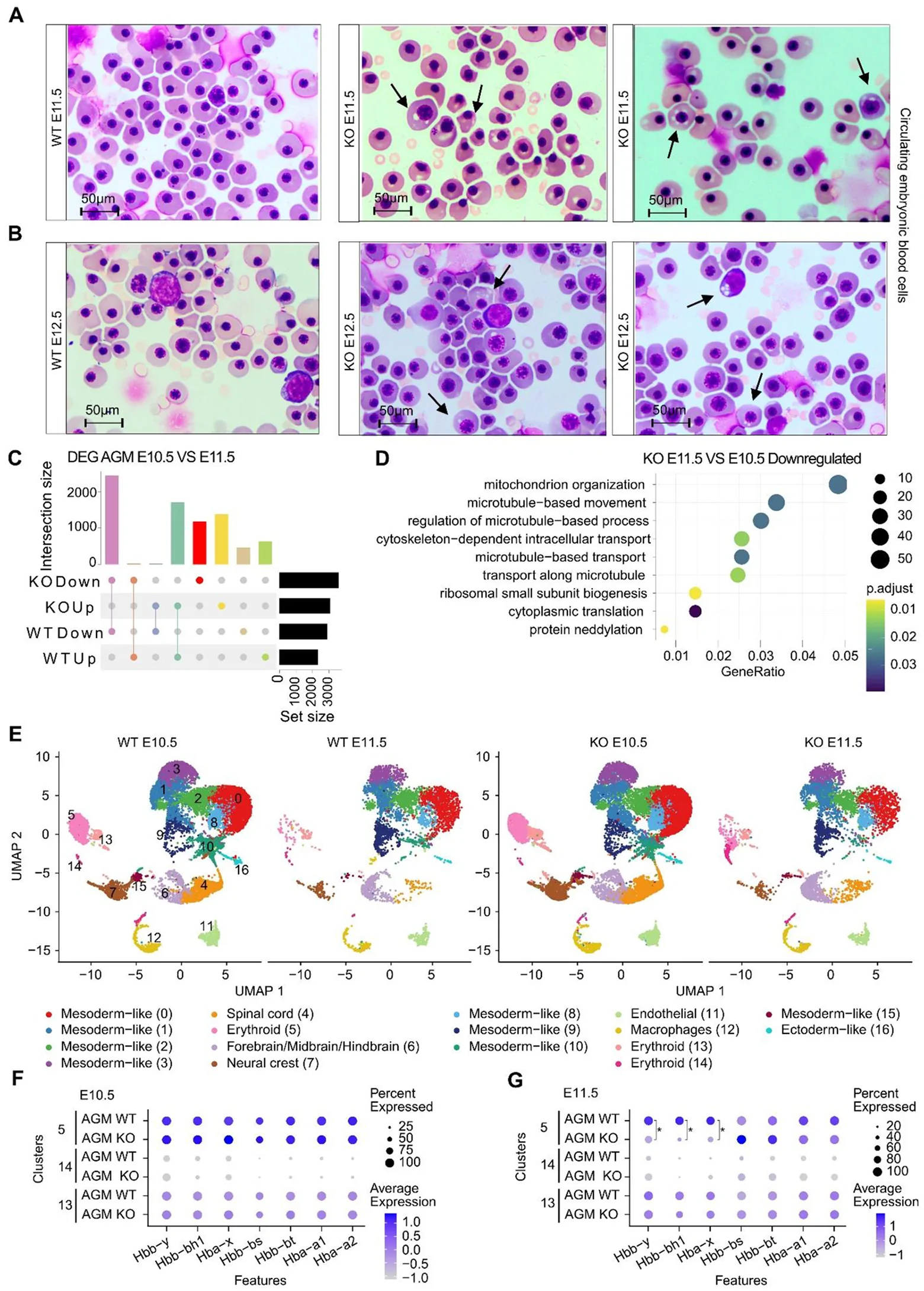
Lack of prostasin affected erythrocyte differentiation in cells detected in the AGM region. **(A, B)** Giemsa staining of circulating erythroid cells isolated from fetal blood of **(A)** E11.5 and **(B)** E12.5 WT and KO embryos; black arrows indicate the presence of cytoplasmatic vacuoles. The images are representative images from three biological replicates. **(C)** Upset plot of differentially down- or upregulated genes in E11.5 compared to E10.5 AGM from KO and WT RNA-seq data (padj < 0.05). Vertical bars show the number of shared and unique genes in each comparison. **(D)** Dot plot showing the enrichment analysis (GO biological processes) of uniquely downregulated genes in the AGM from E11.5 KO embryos compared to the WT. **(E)** UMAP visualization showing annotation of all cell clusters identified by pooled scRNA-seq of AGM from E10.5 and E11.5 WT (left) and KO (right panel) embryos. **(F, G)** Dot plots from scRNA-seq analysis of AGM showing the expression of globin genes in the three erythroid clusters (5,13 and14) at **(F)** E10.5 and **(G)** E11.5 WT and KO embryos. The images are representative of three biological replicates.

The AGM is a highly interactive environment which captures both circulating erythroblasts and hematopoietic progenitors interacting with their specialized microenvironments [47,48]. Thus, to better understand the granularity of this tissue in WT and KO embryos, we performed single cell (sc)RNA-seq of AGM at E10.5 and E11.5. From scRNA-seq data we identified 3 main erythroid populations among 17 clusters found in the AGM. Analysis of globin gene expression revealed that cluster 5 comprised primitive erythroblasts, expressing high level of fetal hemoglobin genes (*Hbb-y* and *Hbb-bh1*) (Fig 5E; S4A Fig). We further identified cluster 13 as multipotent hematopoietic progenitors and cluster 14 as a mixed population of megakaryocyte and erythroid committed progenitors [30,49,50] (S4B and C Figs). Strikingly, primitive erythroid cells in cluster 5 from KO embryos significantly downregulated fetal globin genes at E11.5 suggesting a functional defect (Figs 5F and G). Moreover, we found key silencers of fetal hemoglobin to be significantly upregulated in KO primitive erythroid cells, including *Klf1* and *Sox6* (S4D and E Figs). These data highlighted that in absence of prostasin the downregulation of fetal hemoglobin genes by primitive erythroid cells is likely linked to their intrinsic damage and dysfunction.

### Upregulation of ribosomal protein genes linked to dysfunctional primitive erythroblasts

As the expression of fetal hemoglobin by erythroid progenitors is crucial during primitive erythropoiesis, we evaluated overall differentiation of the AGM-region cells, including the circulating primitive erythroblasts, by entropy analysis of scRNA-seq data. Entropy measures biologically relevant ‘intercellular transcriptomic heterogeneity’ by using the transcriptomic profile of a cell to quantify the relative activation levels of its molecular pathways as defined over an *a priori* specified protein interaction network. Clusters of erythroid progenitor cells exhibited the lowest entropy, especially primitive erythroid cells which are among the earliest fully differentiated cells to form in the mammalian embryo (Fig 6A). Strikingly, at E11.5 we identified a shift of primitive erythroid cells (cluster 5) toward higher entropy in KO embryos (Fig 6B), indicating that this population likely failed to differentiate. These data were in line with the observed downregulation of fetal hemoglobin genes in the KO (Fig 5G). Next, to better understand the effect of *Prss8* depletion on the transcriptomic profiles of distinct cell populations found in the AGM, we performed DEG analysis of scRNA-seq data. Our results revealed that the major change in gene expression occurred at E11.5 in the primitive erythroid cluster 5, with 257 differentially expressed genes (Fig 6C). Most of these genes were found to be upregulated in KO cells. The main upregulated pathways retrieved by GO term analysis were linked to ribonucleoprotein regulation, mainly ribosomal protein small subunit (Rps) and large subunit (Rpl) genes that modulate biological processes including erythrocyte homeostasis and differentiation (Figs 6D and E; S5A Fig). Interestingly, scRNA-seq analysis revealed that *Prss8* was found to be only expressed in ectoderm-like cells, while the other cell clusters including erythroid populations showed no expression (S5B Fig), suggesting again a cell-extrinsic role of prostasin during primitive erythropoiesis.

**Fig 6:**
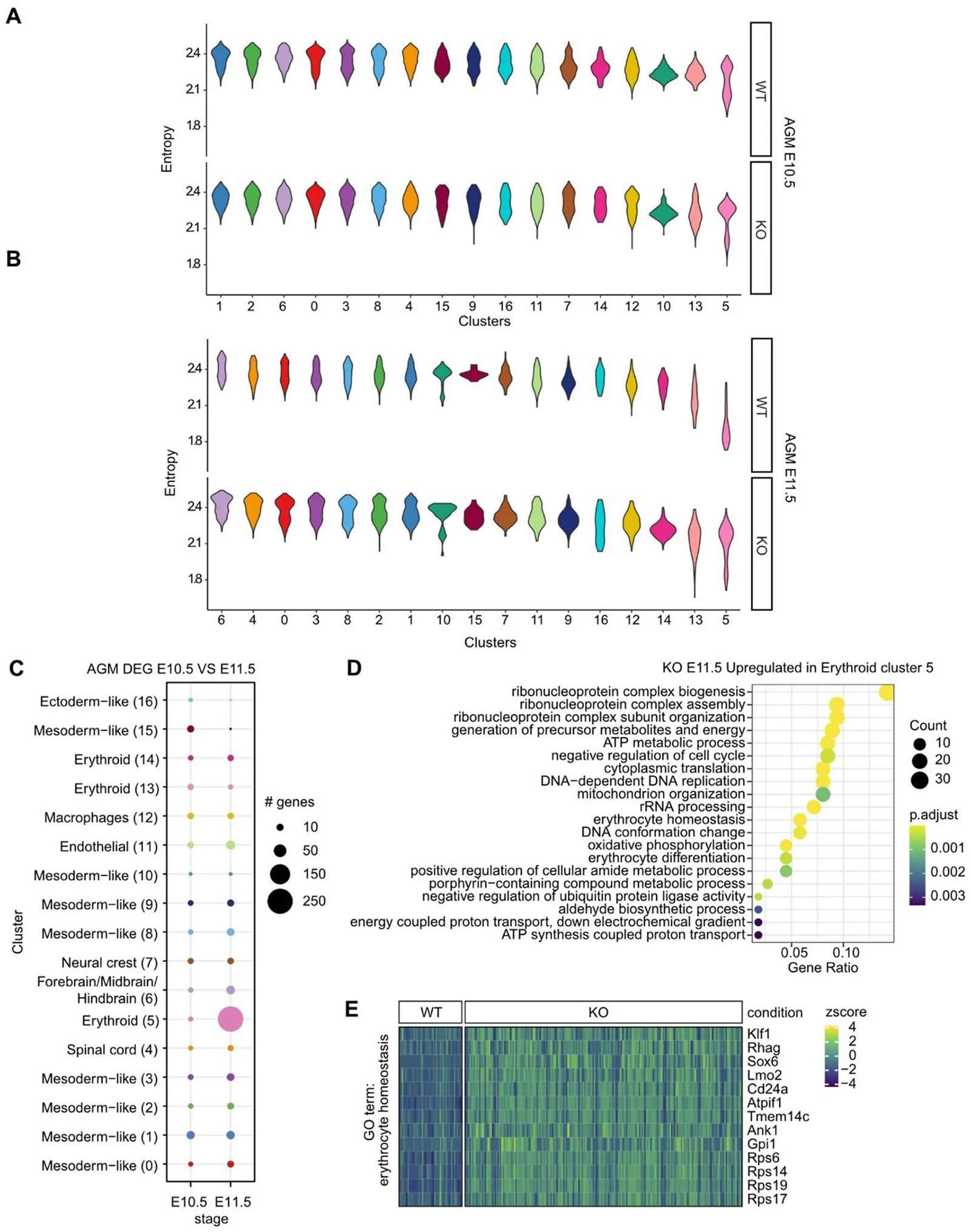
Prostasin deficiency impaired terminal primitive erythropoiesis. **(A, B)** Analysis of cellular entropy of cell clusters identified in the AGM by scRNA-seq from **(A)** E10.5 and **(B)** E11.5 WT and KO embryos. **(C)** Dot plot showing the number of differentially expressed genes (DEG) per cluster in the AGM of E10.5 and E11.5 WT and KO embryos. **(D)** GO Term analysis of upregulated genes in the AGM from E11.5 KO embryos specifically in the erythroid cluster 5. **(E)** Heatmap showing the expression of ‘erythrocyte homeostasis’ related genes.

### Prostasin regulates erythroid maturation but not hematopoietic progenitor generation

Yolk sac-derived erythroblasts further differentiate in the bloodstream and in the fetal liver [30]. We therefore performed imaging flow cytometry in E12.5 fetal livers using markers for Ter119, CD71 and Hoechst. The presence of maturing basophilic (BasoE), polychromatophilic (PolyE) and orthochromic (OrthoE) erythroblasts could be identified in both WT and KO embryos (Figs 7A-D). We detected a significantly reduced frequency of BasoE and PolyE, and particularly of the most differentiated OrthoE in the fetal liver of KO embryos (Fig 7E), indicative of aberrant definitive erythropoiesis. Next, to determine if a general defect in multipotent hematopoietic progenitors exists, the amount of immunophenotypically-defined progenitor cells was analyzed in the yolk sac and AGM at E11.5 by flow cytometry. The frequency of hemogenic endothelium cells (c-Kit^+^; CD45 CD43^-^; CD31Ve-cad^+^) and HSC precursors, both Type-I (CD31^+^CD41^low^CD45^-^CD43^+^cKit^+^) and Type-II (CD31^+^CD41^low^CD45^+^CD43^+^cKit^+^), were comparable in KO and WT embryos [36] (S6A-D Figs). Moreover, mRNA levels of transcription factors that are essential for hematopoiesis in the AGM, such as *Gata1*, *Runx1* and *Tal1*, and of glycoproteins *Epo*, *Epor* were not affected (S6E Fig).

**Fig 7:**
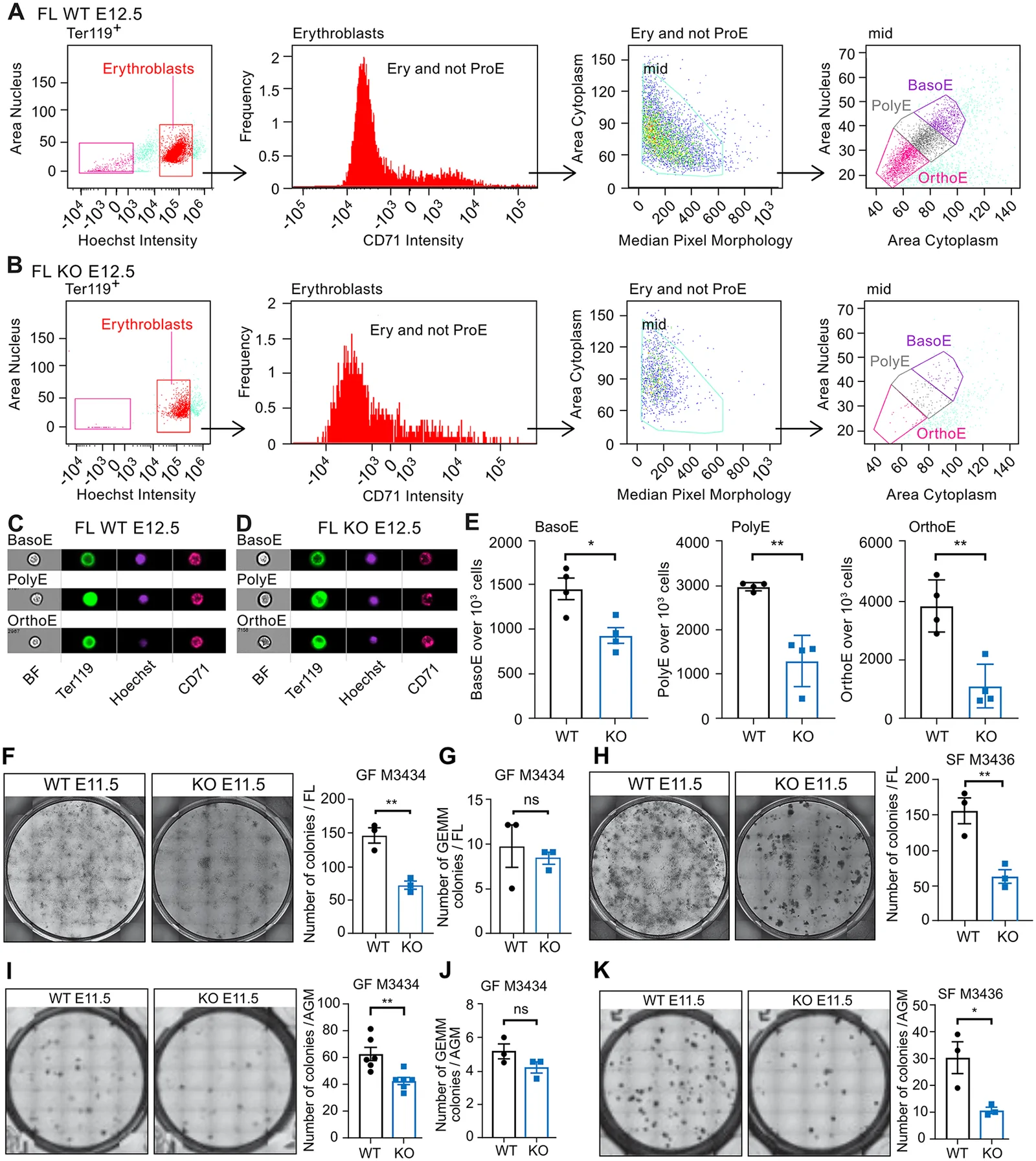
Fetal liver of E12.5 Prss8^-/-^ embryos showed a reduced number of erythroblasts. Flow cytometry analyses of freshly isolated fetal liver (FL)-derived cells from E12.5 **(A)** WT and **(B)** KO embryos. Representative histograms and gating strategy of Ter119 (FITC), CD71 (Pe-Cy7) and Hoechst-stained cells are shown. **(C, D)** Representative images of basophilic (BasoE), polychromatic (PolyE) and orthochromatic (OrthoE) erythroblasts from **(C)** WT and **(D)** KO embryos. Samples were acquired on ImageStreamX imaging flow cytometer (Cytek Biosciences) and analyzed using DIVA acquisition software. **(E)** Quantification of indicated cell populations as cell number over 10^3^ FL cells. Fluorescent signal was recorded with the DIVA acquisition software using FACS Fortessa machine; n = 4. **(F-K)** Colony formation unit (CFU) assays and their quantifications using complete hematopoietic progenitor expansion medium (MethoCult, M3434) on **(F)** fetal liver- and **(I)** AGM – derived cells isolated from E11.5 WT and KO embryos or using selective erythroid progenitor medium (MethoCult, M3436) on **(H)** fetal liver- and **(K)** AGM – derived cells isolated from E11.5 WT and KO embryos. **(G, J)** The largest colonies of multipotent progenitor cells (GEMM colonies, type 4) were identified and quantified from cultures in **(F)** and **(I)** respectively; images taken with StemVision automated microscope (Stem Cell Technologies); n = 3. n: biological replicates (every dot represents one biological replicate as mean of technical replicates). Values in bar plots are presented as the mean ± SEM. Significance was calculated using the unpaired Student t test and all graphs were plotted using GraphPad Prism 8.3.1. *P ≤ .05, **P ≤ .01. The images are representative of three biological replicates.

We further tested hematopoietic progenitor function in CFU assays. *Prss8* KO cells isolated from E11.5 AGM and fetal liver showed overall lower proliferative ability *in vitro* in complete hematopoietic CFU progenitor assays (Figs 7F and I), while the largest colonies of multipotent progenitors that form multilineage colonies of granulocytes, erythrocytes, monocytes, and macrophages (GEMM) were conserved (Figs 7G and J). Despite the overall hematopoietic progenitor defect, in *Prss8* KO AGM and fetal liver cells shown a marked decrease in CFUs in the erythroid-specific CFU assay (Figs 7H and K), suggesting that the defect was mostly driven by a defect in erythroid progenitors. Notably, after FACS analyses of the cells harvested after the M3434 CFU colony assay, we observed a trend toward a higher proportion of CD71 ⁺ erythroid progenitors in KO fetal livers (FL), and a lower proportion of Ter119 ⁺ erythroid cells (S7A-D Figs). These findings suggest that KO cells can commit to the erythroid lineage and generate erythroid progenitors, whereas progression to later stages of erythroid maturation may be compromised. Importantly, myeloid cell maturation appeared largely unaffected (S7C and D Figs). Moreover, we analyzed the amount of lymphoid and myeloid cells present in the freshly isolated fetal livers and we found higher numbers of T cells, dendritic cells (DCs) and neutrophils in KO embryos (S8A-C Figs), even though this difference was not statistically significant. These findings highlighted that *Prss8* deficiency is preferentially implicated in erythroid differentiation, without affecting the production of multipotent progenitor cells or other hematopoietic lineages in murine embryos.

## Discussion

Lack of prostasin is known to cause embryonic lethality, yet its precise biological role during embryonic development remains largely unexplored [24]. Our data revealed a novel function for prostasin in the regulation of primitive erythropoiesis in the developing embryo. We demonstrated that *Prss8* deficiency resulted in transcriptional changes for primitive erythroblasts, which may explain their impaired capacity to terminally differentiate into erythrocytes. The defective erythroid maturation resulted in aberrant remodeling of the yolk sac vasculature, which likely contributed to impaired placental vascularization and lethality of *Prss8*^*-/-*^ embryos at E14.5.

We found the defective vessel remodeling of KO yolk sacs at E12.5 to be associated with a downregulation of key regulators of this pathway at E11.5 (Figs 2A-C; S2A-D Figs). The yolk sac vasculature undergoes major remodeling between E11.5 and E13.5, with a significant decrease of total vessel density due to smaller vessels merging to form large major vessels at E12.5 as a response to the demands of the expanding yolk sac and growing embryo [51]. Therefore, alterations in vascular remodeling became evident in the gross morphology of KO embryos at E12.5, when vascular adaptations to development occur (Figs 2A-C). Interestingly, loss of function for some of the downregulated genes found in the KO embryos namely *Brpf1, Angpt1, Hdac7 and Setd2*, have already been reported to cause embryonic lethality in mice, showing a similar phenotype to *Prss8*^*-/-*^ embryos [52–55]. Although the encoded proteins were described to regulate vessel development, our data suggested that the vascular phenotype observed in yolk sac of *Prss8*^*-/-*^ embryos was not associated with defective vasculogenesis, since blood vessel formation was maintained and embryo development was normal until E11.5 (Figs 2D-F). Instead, we found differences in vessel remodeling and in the frequency of embryonic erythroblasts at E12.5, which were significantly affected in the *Prss8*^*-/-*^ placenta, yolk sac and fetal liver (Figs 3F and 4F and 7E). We reasoned that this difference was tissue-independent because of free cells circulating between extra- and intra- embryonic compartments from E10 onwards [56]. Overall, these findings are in line with other studies showing that erythrocyte content and blood flow influence vessel remodeling [43,44,57]. It remains however unclear whether this defect is a direct consequence of missing prostasin signaling within the hematopoietic niche or represents a secondary effect of the generalized vascular and placenta failure associated with prostasin deficiency. Indeed, the close interplay between placenta function and embryonic growth makes it difficult to unravel primary placenta failure from direct effects on embryonic hematopoiesis. Since prostasin is expressed in both placenta and YS, neither a tissue-specific mechanism nor a synergic contribution of these compartments can be excluded. Interestingly, prostasin dysregulation in the murine epidermis has been reported to induce massive inflammation and premature lethality through downstream PAR2 signaling in a protease-independent manner [58]. Notably, we found *Prss8* and *F2rl1* being expressed by the same cell cluster in the WT YS at E10.5 and E11.5. This data suggests that PAR2-mediated signaling may similarly contribute to the regulation of embryonic erythropoiesis. On the contrary, we believe that ENaC does not play a role here, as *Scnn1g* expression was not detected in embryonic tissues such as YS and AGM at these time points (S1A-C Figs), and deficiency of ENaC does not lead to embryonic lethality [59]. Despite prostasin being a known upstream activator of ENaC in epithelial tissues, where it regulates sodium transport and fluid homeostasis through proteolytic modulation of channel activity, this prostasin–ENaC axis is largely restricted to epithelial systems such as kidney, airways, and skin, and has not been implicated in hematopoietic or early mesoderm-derived compartments. Moreover, the channel (ENaC) is thought to remain closed during fetal life and become only activated at birth [60]

During this developmental window (E10.5-E12.5) different waves of erythropoiesis from the yolk sac (primitive and EMPs-derived erythrocytes) partially overlap. At the same time, the first HSCs begin to colonize the fetal liver and generate the majority of definitive erythroid cells during later stages of mouse gestation [61,62]. In E12.5 KO embryos we report reduced numbers of primitive erythroid cells, the most abundant circulating cells at this timepoint, and of erythroblasts in fetal liver, which likely represent EMP-derived erythroblasts. Additionally, *in vitro* cultures showed that the number of total hematopoietic colonies from AGM and fetal liver was significantly reduced in the KO at E11.5, with conservation of multipotent GEMM colonies in complete methylcellulose CFU assays and a marked defect in erythroid-specific colonies (Figs 7F-K). Furthermore, FACS analysis of CFU colonies showed that erythroid progenitors from KO FL are able to commit to the erythroid lineage and express CD71, although differentiation into Ter119^+^ cells seemed compromised (S7C and D Figs). We thus suggest that the overall reduction in colony number was due to reduced erythroid colony formation. Indeed, immunophenotypically, the frequency of pre-HSCs and EMPs at E11.5 were conserved in the AGM and yolk sac for both genotypes (S6 and S7 Figs). Yet yolk sac derived cells maintained normal colony forming activity (Figs 3G and H), likely because most multipotent and erythroid progenitor cells are mobilized to the bloodstream and fetal liver at this time point [43,50]. Overall, we propose that prostasin is implicated in erythroid maturation but is dispensable for the generation of erythroid progenitors or other hematopoietic cell lineages.

Identification of the erythropoietic niche and characterization of its environment has been the focus of many research studies over the past years. Sampling by scRNA-seq allowed monitoring of these processes with minimal manipulation of the milieu [63,64]. Congruently with our findings, at E10.5 erythroid cell clusters in both KO and WT YS showed similar fetal hemoglobin gene expression. On the contrary, at E11.5 we found their expression to decline in the WT but not in the KO YS (S1C and D Figs), suggesting a compensatory mechanism to sustain primitive erythropoiesis specifically in the YS in response to an erythroid maturation defect. Our scRNA-seq analysis revealed that in the E11.5 AGM, the primitive erythroid cells exhibited lower differentiation features associated with a major change in gene expression, notably the downregulation of fetal hemoglobin genes in the KO (Fig 5G). During erythropoiesis *Hbb-bh1* and *Hbb-y* are known to be upregulated in primitive erythroblasts until E11.5 [29,65]. *Hbb-bh1* and *Hbb-y* downregulation in E11.5 *Prss8*^*-/-*^ AGM region is thus further consistent with impaired erythroblast maturation in absence of prostasin. Moreover, primitive erythroid cells upregulated several genes related to ribonucleoprotein complex regulation, especially *Rpl* and *Rps* genes (Figs 6D and E; S5A Fig), in line with other studies reporting that mutation of ribosomal protein genes was linked to defective erythroid maturation and anemia [66–68]. Importantly, the other two erythroid clusters consisting of mixed erythroid- and megakaryocyte committed progenitors (cluster 14), as well as multipotent hematopoietic progenitors (cluster 13) did not reveal any major transcriptional differences nor differentiation defect (Fig 6C). Furthermore, we observed the presence of cytoplasmatic vacuoles in basophilic and polychromatic erythroblasts in the circulation of E11.5 and E12.5 KO embryos (Figs 5A and B), a feature associated with cellular stress and cell death [69–71]. Clathrin-dependent vacuole generation in erythroblasts contributes to nuclear extrusion and thus defective erythroblast maturation [72], further suggesting that primitive erythroid precursors are generated *in vivo* but may fail to complete terminal maturation in the absence of prostasin. In line with this, RNA-seq data analysis from AGM of E11.5 *Prss8*^*-/-*^ embryos identified a set of downregulated genes related to microtubule and cytoskeleton complex organization (Figs 5C and D). These processes are known to be critical during late stages of terminal erythropoiesis [46,68,73], further supporting a defect in terminal primitive erythropoiesis upon loss of prostasin. In addition, general defects in the endosome/lysosomal trafficking pathway during embryogenesis have been associated with the accumulation of erythroblastic intracytoplasmic vacuoles, including autophagosomes [73]. Similar erythroblast vacuolization is observed in the adult bone marrow of patients with VEXAS syndrome [74], although the underlying mechanisms may differ. Further investigation will be required to determine the nature of the vacuoles observed in *Prss8*^−/−^ erythroblasts and to establish whether altered vesicular trafficking contributes to their formation and to the associated erythroid phenotype.

Interestingly, we found prostasin to be only expressed in the yolk sac from E9.5 until E12.5, coinciding with the site of primitive erythropoiesis. Contrarily, no -or at most scarce- expression of prostasin was detectable in circulating primitive erythroid cells (Fig 1), suggesting that prostasin likely controls primitive erythropoiesis in a cell-extrinsic manner. Congruently, our scRNA-seq data found *Prss8* to be mostly expressed by non-hematopoietic cells (S5B Fig). Considering that prostasin can be released from the cell membrane to the surrounding space and affect cells in other tissues or organs [75–77], we speculate that prostasin may control terminal erythropoiesis via protein-protein interaction with known binding partners such as PAR2 [14,58,78] or other serine proteases, such as matriptase [75]. On the other hand, it has been reported that *Prss8* is expressed by late erythroid cells in E13.5 murine and human fetal liver [79,80], suggesting that prostasin expression may be induced during definitive erythropoiesis in those cells. Indeed, two erythroid-speciﬁc promoter elements have been identified in the *Prss8* gene to function in a cell-intrinsic manner [81]. These hypotheses require further investigations and *in-vivo* models with cell specific deletion as well as co-culture experiments will help to further unveil the role of *Prss8* during distinct fetal erythropoietic waves.

In conclusion, while the main target of prostasin still needs to be identified, our data highlights that *Prss8* loss impairs murine primitive erythroid maturation between E11.5 and E12.5, and manifests through an erythroid-specific maturation defect, alteration in fetal hemoglobin gene expression and upregulation of *Rps* and *Rpl* genes. This warrants further studies on prostasin and may provide novel insights for erythropoiesis-related disorders. Moreover, the developmental defect of the placental vasculature at E14.5 associated with *Prss8*^*-/-*^ embryonic lethality [23,24] and onset of preeclampsia associated with *Prss8* polymorphism [23,24] may indeed occur as a consequence of reduced embryonic erythropoiesis.

## Supporting information

S1 Fig Sequencing analysis of the YS from E10.5 and E11.5 WT and KO embryos. **(A)** UMAP visualization showing annotation of all cell clusters identified by pooled scRNA-seq of YS from E10.5 and E11.5 WT and KO embryos. **(B)** Dot plot from scRNA-seq analysis showing *Prss8, F2rl1 (*encoding PAR2*) and Scnn1g* expression in the distinct cell clusters of the YS from E11.5 WT embryos. **(C,D)** Dot plots from scRNA-seq analysis of YS showing the expression of globin genes in the three erythroid clusters (16,10 and 1) at **(C)** E10.5 and **(D)** E11.5 WT and KO embryos.
(TIF)

S2 Fig RNA-seq analyses of the yolk sac from E10.5 and E11.5 WT and KO embryos. **(A, D)** Heatmaps showing the expression levels of differentially expressed genes (DEG) related to **(A, D)** angiogenesis and **(B, C)** vasculogenesis comparing **(A, B)** E10.5 and E11.5 for each genotype, and **(C, D)** comparing WT and KO embryos for each timepoint.
(TIF)

S3 Fig Expression of hypoxia related genes in AGM and yolk sac at E11.5. **(A, B)** Heatmaps showing the expression of ‘response to hypoxia’ related genes in E11.5 **(A)** AGM and **(B)** yolk sac of WT and KO embryos from bulk RNA-seq analysis.
(TIF)

S4 Fig Three main erythroid cell clusters identified in the AGM are identified by scRNA-seq. **(A)** Violin plot of representative gene markers used to identify cells clusters in AGM samples of scRNA-seq data. **(B, C)** Dot plots of representative markers used to identify erythroid clusters of **(B)** pre-HSCs and **(C)** mixed erythroid and megakaryocyte progenitor cells. **(D, E)** Dot plots of fetal hemoglobin activators and inhibitor gene expression in erythroid clusters at **(D)** E10.5 and **(E)** E11.5 embryos. * p adjusted value < 0.05.
(TIF)

S5 Fig Sequencing analysis of the AGM from E10.5 and E11.5 WT and KO embryos. **(A)** Heatmap showing the expression of significantly upregulated genes related to ‘Ribonucleoprotein-related pathways’ in cluster 5 from the E11.5 AGM (scRNA-seq analysis)**. (B)** Dot plot from scRNA-seq analysis showing *Prss8* expression in the distinct cell clusters of the AGMs from E11.5 WT embryos.
(TIF)

S6 Fig Gating strategy for HSCs and gene expression of erythropoiesis genes in E11.5 AGM. **(A, B)** Representative plots of flow cytometry analysis using antibodies against CD45 (BUV 737), CD41 (FITC), CD43 (PE7Cy7) and CD31 (PE) on AGM-derived cells from E11.5 **(A)** WT and **(B)** KO embryos identifying Type I and Type II pre-HSC cells; same FACS axis for gating 1 and 2; fluorescent signal was recorded with the DIVA acquisition software using FACS Fortessa machine. **(C,D)** Quantification of **(C)** Type I and Type II pre-HSC cells and **(D)** hemogenic endothelium from yolk sac (YS) and AGM-derived cells expressed as cell number over 10^3^ total cells; n = 4. **(E)** Heatmap showing the expression levels of indicated genes in the AGM from E10.5 and E11.5 WT and KO embryos. n: biological replicates (every dot represents one biological replicate). Values in bar plots are presented as the mean ± SEM. Significance was calculated using the unpaired Student t test and all graphs were plotted using GraphPad Prism 8.3.1.
(TIF)

S7 Fig Gating strategy for myeloid and lymphoid cells in CFUs from E12.5 fetal liver. Single fetal liver cells were isolated from WT and KO and plated for CFU assays on day 0. **(A)** Representative plots of flow cytometry analysis for hematopoietic cells recovered from CFU colonies at day 10 after plating using antibodies against CD71, Ter119 (BV711), NK-1 (FITC), CD11b (BV421), CD11c (PerCP/Cy5.5), F4/80 (PE-Texas red) and Ly6G (APC/Cy7). Fluorescent signal was recorded with the DIVA acquisition software using FACS Fortessa machine. (**B)** Quantification of total colony formation units (CFU) using complete hematopoietic progenitor expansion medium (MethoCult, M3434) on fetal liver-derived cells isolated from E12.5 WT and KO embryos. **(C,D)** Quantification of **(C)** the percentage of myeloid (GM) and erythroid (CD71+) progenitors, and Ter119 + cells; **(D)** the mean fluorescent intensity of the indicated markers. n: biological replicates (every dot represents one biological replicate). Values in bar plots are presented as the mean ± SEM. Significance was calculated using the unpaired Student t test and all graphs were plotted using GraphPad Prism 8.3.1.
(TIF)

S8 Fig Gating strategy for myeloid and lymphoid cells in E12.5 fetal liver. **(A,B)** Representative plots of flow cytometry analysis using antibodies against CD45 (BUV737), B220 (BV711), NK-1 (FITC), CD11b (BV421), CD11c (PerCP/Cy5.5), F4/80 (PE-Texas red) and Ly6G (APC/Cy7) on cells isolated from **(A)** WT and **(B)** KO embryos. Fluorescent signal was recorded with the DIVA acquisition software using FACS Fortessa machine. **(C)** Quantification of indicated cell types expressed as cell number over 10^3^ total cells from WT and KO embryos; n = 3. n: biological replicates (every dot represents one biological replicate). Values in bar plots are presented as the mean ± SEM. Significance was calculated using the unpaired Student t test and all graphs were plotted using GraphPad Prism 8.3.1.
(TIF)

S9 Fig Raw_images. Original uncropped and unadjusted images of western blot membrans for **(A)** Fig.1B, **(B)** Fig.1C and **(C)** Fig.1E.
(TIFF)

S1 Appendix Supplementary methods. (DOCX)
