# Supplementary material for: Impaired erythroid maturation in murine embryos upon loss of the preeclampsia-associated serine protease prostasin Prostasin loss impairs erythropoiesis
Source: PLoS One. 2026 Oct 16;21(10):e0360958. doi: 10.1371/journal.pone.0360958 (PMC13674711; doi:10.1371/journal.pone.0360958)
Supplement: Supplementary file 1 [file pone.0360958.s010.docx]

**Supporting information**

**Supplementary methods**

**Bulk RNA-sequencing analyses**

For data analysis**,** FASTQ files were aligned to the mouse genome annotation (GRCm39 release 105, retrieved from Ensembl database) using STAR algorithm (version 2.7.10a) (Dobin et al., 2013). The output count matrices were imported in R (version 4.0.2). AGM and yolk sac data sets were analyzed separately. Analysis of the differentially expressed genes (DEGs) was performed with DESeq2 (version 1.21.1) (Love et al., 2014) and p adjusted value of < 0.05 was considered significant. Heatmap representations were made with the ComplexHeatmap package (version 2.4.3) using normalized counts as input. DEGs analysis for the bulk-RNA seq data was performed independently for each genotype comparing time points (E10.5 vs E11.5). The list of DEGs (p adjusted value < 0.05) was divided in upregulated (log2 fold change > 0) and downregulated (log2 fold change < 0) gene sets and subsequently combined in a binary matrix. Uniquely and shared gene sets were displayed as an UpSet plot.

Functional analysis was performed with the over-representation test function from ClusterProfiler package (version 3.16.1) (Yu et al., 2012), selecting biological processes as the ontology and using as input specific gene sets derived from the UpSet plots. GO terms were considered significant using a threshold of p adjusted value < 0.05. Heatmap representations for selected GO terms or gene sets were made with the ComplexHeatmap package (version 2.4.3) using normalized counts as input.

**scRNA-sequencing data analyses AGM and bioinformatics**

Fastq files were processed with CellRanger count with the default settings (cellranger-7.0.1) on the mouse genome reference refdata-gex-mm10-2020-A. Count matrices were imported into R (version 4.0.2) using the Seurat package (version 4.0.1) (Satija et al., 2015). Cell filtering was performed by applying sample specific thresholds after exploratory analysis. More specifically, cells with more than 1000 (AGM E10.5 dataset) or 1500 (AGM E11.5 dataset) and less than 7000 features, and mitochondrial percentage lower than 10% were kept for further analysis. Both Seurat objects were then combined using RPCA integration. Cell type annotation was performed with singleR package (version 1.2.4) (Aran et al., 2019) using as reference a subset of the scRNA seq dataset derived from mouse gastrulation and early organogenesis. (Pijuan-Sala et al., 2019) More specifically we used the set derived from the E10.5 stage, the latest stage present in the dataset and the closest to the time points included in our experiment. Additionally, the automatic cell annotations were manually adjusted and refined based on reported gene markers (Lummertz da Rocha et al., 2022). Analysis of the three erythroid cluster genes was performed using as reference a list of murine genes retrieved from three different publications on E9.5 and E10.5 yolk sac and E12.5 and E13.5 fetal liver single cell landscape (Ceccacci et al., 2023; Iturri et al., 2021; Soares-Da-Silva et al., 2021). DEG analyses were performed using Seurat (version 4.2.0) (Butler et al., 2018) built-in functions (FindConservedMarkers, FindAllMarkers). Moreover, differentially expressed genes with a p adjusted value <0.05 were considered significant and used as input for other downstream analyses as required. Functional analysis of gene sets was performed with the over-representation test function from ClusterProfiler (version 3.16.1) selecting biological processes as the ontology. Heatmap representations for selected GO terms or gene sets were made with the ComplexHeatmap package (version 2.4.3) using as input normalized counts.

For entropy estimation each tissue data set was divided by time point, while keeping both genotypes in each subset. Single cell entropies were computed using the SLICE algorithm (version 0.99.0)(Guo et al., 2017) and the results were visualized with ggplot2 (version 3.3.3).

**scRNA-sequencing data analysis Yolk Sack** **and bioinformatics**

Fastq files were processed with CellRanger count with the default settings (cellranger-7.0.1) on the mouse genome reference refdata-gex-mm10-2020-A. Count matrices were imported into R (version 4.0.2) using the Seurat package (version 4.0.1) (Satija et al., 2015). Cells filtering was performed by applying sample specific thresholds after exploratory analysis. More specifically, cells with more than 900 (YS E10.5 dataset) or 500 (YS E11.5 dataset) and less than 7000 features, and mitochondrial percentage lower than 20% were kept for further analysis. Both Seurat objects were then combined using RPCA integration.

Cell type annotation was performed with singleR package (version 1.2.4) (Guglani et al.) using as reference a public scRNAseq dataset derived from mouse Yolk Sack sorted populations (Biben et al., 2023).

**Supplementary References**

**Aran, D., Looney, A. P., Liu, L., Wu, E., Fong, V., Hsu, A., Chak, S., Naikawadi, R. P., Wolters, P. J., Abate, A. R., et al.** (2019). Reference-based analysis of lung single-cell sequencing reveals a transitional profibrotic macrophage. *Nat. Immunol.* **20**, 163–172.

**Biben, C., Weber, T. S., Potts, K. S., Choi, J., Miles, D. C., Carmagnac, A., Sargeant, T., de Graaf, C. A., Fennell, K. A., Farley, A., et al.** (2023). In vivo clonal tracking reveals evidence of haemangioblast and haematomesoblast contribution to yolk sac haematopoiesis. *Nat. Commun.* **14**,.

**Butler, A., Hoffman, P., Smibert, P., Papalexi, E. and Satija, R.** (2018). Integrating single-cell transcriptomic data across different conditions, technologies, and species. *Nat. Biotechnol.* **36**, 411–420.

**Ceccacci, E., Villa, E., Santoro, F., Minucci, S., Ruhrberg, C. and Fantin, A.** (2023). A Refined Single Cell Landscape of Haematopoiesis in the Mouse Foetal Liver. *J. Dev. Biol* **15**,.

**Dobin, A., Davis, C. A., Schlesinger, F., Drenkow, J., Zaleski, C., Jha, S., Batut, P., Chaisson, M. and Gingeras, T. R.** (2013). Sequence analysis STAR: ultrafast universal RNA-seq aligner. *Bioinformatics* **29**, 15–21.

**Guglani, L., Lakshminrusimha, S., Ryan, R. M. and Guglani, D.** *Transient Tachypnea of the Newborn*.

**Guo, M., Bao, E. L., Wagner, M., Whitsett, J. A. and Xu, Y.** (2017). SLICE: determining cell differentiation and lineage based on single cell entropy. *Nucleic Acids Res.* **45**, 1–14.

**Iturri, L., Freyer, L., Biton, A., Dardenne, P., Lallemand, Y. and Gomez Perdiguero, E.** (2021). Megakaryocyte production is sustained by direct differentiation from erythromyeloid progenitors in the yolk sac until midgestation. *Immunity* **54**, 1433–1446.

**Love, M. I., Huber, W. and Anders, S.** (2014). Moderated estimation of fold change and dispersion for RNA-seq data with DESeq2. *Genome Biol.* **15**, 1–21.

**Lummertz da Rocha, E., Kubaczka, C., Sugden, W. W., Najia, M. A., Jing, R., Markel, A., LeBlanc, Z. C., dos Santos Peixoto, R., Falchetti, M., Collins, J. J., et al.** (2022). CellComm infers cellular crosstalk that drives haematopoietic stem and progenitor cell development. *Nat. Cell Biol.* **24**, 579–589.

**Pijuan-Sala, B., Griffiths, J. A., Guibentif, C., Hiscock, W., Jawaid, W., Calero-Nieto, F. J., Mulas, C., Ibarra-Soria, X., Tyser, R. C. V, Lee Lian Ho, D., et al.** (2019). A single-cell molecular map of mouse gastrulation and early organogenesis. *Nature* **566**, 490–495.

**Satija, R., Farrell, J. A., Gennert, D., Schier, A. F. and Regev, A.** (2015). Spatial reconstruction of single-cell gene expression data. *Nat. Biotechnol.* **33**, 495–502.

**Soares-Da-Silva, F., Freyer, L., Elsaid, R., Burlen-Defranoux, O., Iturri, L., Sismeiro, O., Pinto-Do, P., Gomez-Perdiguero, E. and Cumano, A.** (2021). Yolk sac, but not hematopoietic stem cell–derived progenitors, sustain erythropoiesis throughout murine embryonic life. *Journal of Experimental Medicine* **218**, 1–16.

**Yu, G., Wang, L. G., Han, Y. and He, Q. Y.** (2012). ClusterProfiler: An R package for comparing biological themes among gene clusters. *OMICS* **16**, 284–287.
