# Supplementary material for: Impaired erythroid maturation in murine embryos upon loss of the preeclampsia-associated serine protease prostasin Prostasin loss impairs erythropoiesis
Source: PLoS One. 2026 Oct 16;21(10):e0360958. doi: 10.1371/journal.pone.0360958 (PMC13674711; doi:10.1371/journal.pone.0360958)
Supplement: Supplementary file 2 [file pone.0360958.s011.docx]

PONE-D-26-08825R1

**Title :** Impaired erythroid maturation in murine embryos upon loss of the preeclampsia-associated serine protease prostasin

**Response to Reviewers**

We would like to thank the reviewers for their constructive comments and suggestions. New data were further analyzed, and text was amended as suggested. Following specific changes were made:

- Additional analyses of data were performed and included as new figures (SFig1 for scRNAseq data on YS, SFig7 for FACS analysis or erythroid colony output in CFU assays)
- Fig.3 was modified as proposed
- The protocol for the Western blot analysis was introduced in the Methods section.
- The protocol of the new scRNA-seq analyses of the YS was added as Supplementary information.
- Reference 43 was corrected.
- Results and discussion were amended as proposed.

**Reviewer #1:** In this study, the authors identified defective erythroid maturation in Prss8-/- embryos at E11.5 and E12.5 in the yolk sac, placenta, and fetal liver. They propose that prostasin, encoded by the Prss8 gene, regulates primitive erythropoiesis, despite its absence in erythroid cells during this developmental window. Instead, they observed impaired vascular remodeling in the yolk sac of Prss8-/- embryos. Based on these findings, they suggest that prostasin plays a previously unrecognized role in developmental erythropoiesis and in vascular patterning in both the placenta and embryo.

This is a well executed, technically sound, and interesting study by a research group that has long been engaged in prostasin research. The data are convincing, and the study provides valuable insights. This reviewer has several comments that may further strengthen the manuscript.

1) In Fig. 1, the authors show distinct prostasin expression in the yolk sac tissue. However, it remains unclear which specific cell types express this protein in the yolk sac. Immunohistochemical analysis or in situ hybridization to identify prostasin expressing cells would greatly enhance the manuscript.

Animal experimentation is tightly regulated and controlled by local and Swiss authorities. Due to the given timeline for the revision, we could not perform further yolk sac analysis, but we have now addressed this issue through sc-RNA data analyses. At both E10.50 and E11.5, we found *Prss8* being mostly -if not exclusively- expressed within the extra-embryonic (EXE) endoderm cluster. This is now included in the manuscript as new SFig.1 and discussed in line 239 to 243.

2) It is plausible that yolk sac endothelial cells, as well as endothelial cells in the placenta and liver sinusoids, express prostasin and thereby support developmental erythropoiesis. This reviewer strongly recommends examining prostasin expression in endothelial cells. If endothelial cells indeed express prostasin, co culture experiments using Prss8-deficient or wild-type endothelial cells with HSCs or erythroblasts would be highly informative.

We thank the reviewer for this interesting point. Due to local and Swiss regulation for animal experimentation, and the given timeline for the revision, co-culture experiments were not feasible, and might be subject of a follow-up study. However, our scRNA-seq analysis of the yolk sac showed that endothelial cells do not express *Prss8.* This is now included in the manuscript as SFig.1.

3) Do erythroid cells express PAR2?

We now analyzed PAR2 gene expression (*F2rl1*) in the yolk sac using our scRNA-seq data and found that PAR2 is not expressed by erythroid cells, but mainly if not exclusively by the extraembryonic endoderm (EXE) cluster at E10.5 or E11.5, interestingly overlapping with *Prss8* expression. This is now included in the manuscript in SFig1.

4) It would be interesting to know whether the observed phenotype depends on the protease activity of prostasin. Please discuss this point.

This is an interesting point; however, due to the small embryo size as well as the problem of specifically monitoring prostasin activity, this will be a difficult task. We now further discuss this possibility in the manuscript lines 491 to 502.

**Reviewer #2:** The study by Di Carlo et al. investigates the role of the membrane-bound serine protease prostasin (encoded by Prss8) during murine embryonic development. Prss8 deficiency leads to embryonic lethality around E14.5. The authors previously demonstrated that these embryos show impaired placental labyrinth maturation. They now demonstrate that Prss8 deficient embryos also show defective vascular remodeling in the yolk sac. A significant finding is the reduced frequency of both primitive and definitive erythroblasts in the circulation and hematopoietic tissues (yolk sac, placenta, and fetal liver) at E11.5 and E12.5. Single-cell RNA sequencing (scRNA-seq) of WT and Prss8 deficient AGM regions reveals that Prss8-/- primitive erythroid cells have a unique transcriptomic profile, including the downregulation of fetal globin genes and the upregulation of ribosomal protein genes. Because prostasin is not expressed in the erythroid cells themselves, the authors conclude it regulates erythropoiesis through a cell-extrinsic mechanism likely linked to the vascular niche. Overall, the study is well-conceived and provides valuable insights into the non-cell-autonomous role of prostasin in erythropoiesis; however, I have several concerns, which should be addressed before the manuscript is suitable for publication.

Major points
1. The authors suggest that prostasin regulates erythropoiesis in a cell-extrinsic manner because the protease is expressed in the vasculature but not in erythroid cells. However, it remains unclear whether the erythroid maturation defect is a direct consequence of missing prostasin signaling in the niche or a secondary effect of the generalized vascular and placental failure (e.g., hypoxia or nutrient deficiency). The manuscript would benefit from a more detailed discussion or additional experimental evidence (such as in vitro co-culture) to decouple these two phenotypes.

We agree that it would be interesting to follow this in more detail. Due to Swiss and local regulation for animal experimentation, and the given timeline for the revision, such experiments are now not feasible and should certainly be the subject of a follow-up study. We now further discuss these possibilities in the manuscript (lines 485 to 491).

2. While a cell-extrinsic role is proposed, the manuscript does not identify the specific target(s) of prostasin in this context. Given that prostasin is known to activate the epithelial sodium channel (ENaC) and Protease-Activated Receptor 2 (PAR2) in other tissues, the authors should discuss whether these pathways are active in the embryonic vascular niche and if their dysregulation could explain the observed phenotype.

As mentioned above as comment to Reviewer 1, we have now analyzed PAR2 gene expression in the yolk sac through scRNA-seq data and have found that *F2rl1* (encoding for PAR2) is expressed mainly if not exclusively by the extraembryonic endoderm (EXE) cluster, interestingly overlapping with *Prss8* expression. ENaC (alpha subunit) *Scnn1g* is not expressed in the yolk sac, therefore suggesting that the Prss8-ENaC axis may not play a role in Prss8-driven embryonic lethality. This is now included in SFig1 and further discussed in the manuscript (lines 491 to 502).

3. The authors conclude that embryonic lethality is "presumably due -at least in part- to anemia". However, the described defects in the placental labyrinth and yolk sac vascular remodeling are often independently sufficient to cause lethality. A more critical comparison of the severity of the anemia versus the placental failure would be needed to support the claim that anemia is a major driver of the lethality.

We agree with the reviewer and have modified the text accordingly.

4. While the study includes an interesting analysis of globins in the AGM (Fig. 5), a parallel analysis in the yolk sac is missing. Since the YS appears to be the only tissue expressing Prss8, it is crucial to investigate the globin status in this district. If scRNA-seq data is unavailable for this tissue, the authors should perform bulk qPCR or analysis on sorted cell populations to confirm whether this phenotype is also present in the protein's primary site of expression.

We newly analyzed the expression of globin genes through scRNA.seq analyses of WT and KO YS at E10.5 and E11.5, and found no decline for any fetal globin genes across erythroid clusters in the KO (SFig1 C-D). In comparison, those genes were strongly reduced in the KO AGM at E11.5 (Fig 5G). This suggests that *Prrs8* influences erythroblast migration and/or further differentiation rather than the generation of primitive erythroid progenitors in the yolk sac. This is now included in SFig.1 and further discussed in the manuscript lines 521 to 524.

5. In several key experiments (e.g., Fig. 1A and Fig. 3G, H), the number of biological replicates appears somewhat limited. While results are reported as non-significant, the data trends suggest that increasing the sample size might reveal statistically significant differences. If possible, the authors could increase the number of replicates for these experiments to avoid false negatives and strengthen their conclusions regarding Prss8 function.

Due to local and Swiss regulation on animal experimentation, and the given timeline for the revision, repetition of such experiments is now not feasible. For Fig. 1A, we have now reformulated the conclusion more carefully. In Fig.3G,H, there is a suggested increased number of colonies in KO YS-derived cultures, and this might likely be due to the difference between progenitor activity in vitro and in vivo. It is plausible that erythroid progenitors accumulate and that terminal differentiation into erythrocytes is defective in the KO. This is now mentioned in the manuscript.

6. The Materials and Methods section lacks details regarding the Western Blotting. It is necessary to specify the amount of protein loaded per well (µg), as well as full details for the antibodies used (catalog number, supplier, and dilution) to ensure reproducibility.

We apologize for any missing information and have now included all the details in a paragraph of “Material and methods” section.

7. The data show the absence of Prss8 protein in the E9.5 embryo and the E11.5 fetal liver (FL) or AGM. However, no Western Blotting analysis of the placenta is included. Given the embryonic lethality linked to placental defects, placenta (or specifically labyrinth) data should be included.

We previously analyzed the mRNA transcription profile of murine Prss8 in the placenta of E11.5 – E16.5 embryos (PMID: 23405214) and found a peak of expression in E13.5 WT embryos. This increase is missing in Prss8 KO embryos and thus precedes the failure of placental maturation underlining a time-dependent requirement of Prss8 expression during mouse embryogenesis. In our study, we focused on earlier embryonic time-points (E9.5 – E11.5) that revealed an impaired erythroid maturation in the AGM and fetal liver in Prss8 KO embryos before the placental failure becomes evident. Currently, we cannot exclude the contribution of placenta failure to this phenotype, but this will certainly be subject of a follow-up study. The issue is now discussed in lines 486-491 of the manuscript.

8. The decrease in CFU in the AGM and FL shown in Fig. 7H and K is attributed to a decrease in erythroid colonies, although these have not been scored separately in the M3434 assay. Can the authors provide data showing the number of erythroid colonies separately here? Also in this paragraph (lines 389-399) the authors seem to confuse the “complete hematopoietic CFU progenitor assays” (Figs 7F and I), as these are actually shown in Fig. 7G-K. Vice versa for the erythroid-specific CFU assay, which is not shown in Figs 7H and K as indicated but in Fig. 7F-I.

We thank the reviewer for this point. Since the StemCell Vision linked method does not allow for the reliable separation of erythroid colonies in the M3434 medium conditions, we have performed FACS analyses of FL derived cells after 7 days of culture to evaluate erythroid lineage differentiation on a single cell level after CFU assays. The new data is now presented as new SFig.7 and discussed in lines 432 to 440 of the manuscript. We have also corrected the order of panels on Fig. 7.

Minor points
1. Lines 213-214: “Vascular quantification at E12.5 revealed a significantly reduced number of vessel branches and junctions and a lower maximum branch length (Fig 2C).” This statement is not supported by the data: the graph in 2C shows no difference in maximum branch length but instead shows a reduction in the junctions. Moreover, the authors claim that the defective vascular phenotype of the KO was not evident at E11.5 but Figure 2E does show smaller arteries in the KO. I would suggest performing the vascular quantification analysis at E11.5 (and possibly at E10.5 as well) as additional time points to confirm the timing of the onset of the vascular remodeling defects.

We thank the reviewer for raising this point. We have now corrected the sentence accordingly. We conclude that the vascular defect became evident at E11.5 based on multiple complementary analyses, namely gross morphology (Fig.2A), YS immune fluorescence (IF) (Fig.2B), and bulk RNA sequencing DEG analysis between E10.5 and E11.5 (Fig.2I), all of which consistently support a vascular defect at E11.5. Therefore, we concluded that additional analyses of E10.5 YS IF would not further strengthen this point.

2. Lines 256-258: “H&E staining of the yolk sac at E12.5 revealed the presence of a vast majority of nucleated erythroblasts within vessels of WT embryos and significantly fewer nucleated erythroblasts in the KO (Fig 3A)” No statistical test was performed to evaluate statistical significance here, so this statement should be changed. Moreover, the images shown in Fig 2A appear to show two different regions of the YS. Arteries are visible in the WM image presented in Figure 2B so it should be possible to identify similar regions.

We have changed the text accordingly.

3. I would suggest showing individual channels in Fig. 3B for clarity.

We have now modified Fig.3B accordingly and included images for each channel.

4. The presence of cytoplasmic vacuoles in Prss8-/- erythroblasts is noted as a key morphological defect. The authors should comment on whether these vacuoles represent autophagic vesicles, degenerating organelles, or another form of cellular stress.

We thank the reviewer to raise this point. We have now discussed this in the manuscript lines 537 to 549.

5. Page 3, lines 69-73: Both the erythrocytes derived by the EMP wave and the third wave are referred as “definitive”. This can be confusing and the wording in this section should be changed. Also, to my knowledge there is no conclusive evidence that bona fide HSCs actually emerge in the YS.

We have now changed the text accordingly and refer to EMP-derived erythrocytes and HSC-definitive erythrocytes for better clarity.

6. Page 14, line 377: reference 43 appears to be incorrect.

We thank the reviewer for noticing this mistake. We now refer to the correct reference.
