# Supplementary material for: Impaired erythroid maturation in murine embryos upon loss of the preeclampsia-associated serine protease prostasin Prostasin loss impairs erythropoiesis
Source: PLoS One. 2026 Oct 16;21(10):e0360958. doi: 10.1371/journal.pone.0360958 (PMC13674711; doi:10.1371/journal.pone.0360958)
Supplement: Supplementary file 3 [file pone.0360958.s012.docx]

PONE-D-26-08825R1

**Title :** Impaired erythroid maturation in murine embryos upon loss of the preeclampsia-associated serine protease prostasin

**Response to Reviewers**

We would like to thank the reviewers for their constructive inputs and for giving us the chance to improve our manuscript. The text in results and discussion was amended as suggested.

**Review Comments to the Author**
Reviewer #1: The manuscript has been partially improved in response to my comments. Although a few issues remain to be addressed, I accept the authors’ rebuttal and have no further comments. Thank you for your responses.

We thank the reviewer that helped us improving our manuscript.

Reviewer #2: In the revised manuscript, the authors have addressed my concerns by adding new analyses, amending claims and figures and expanding the discussion.

Specifically, they have now incorporated new scRNAseq data analysis of the YS (SFig 1), which shows that Prss8 is expressed in the extra-embryonic endoderm (EXE), though they see no decrease in fetal globins in the YS. Also, PAR2 appears to be expressed by the EXE. In the absence of any new functional data, these transcriptomic data and relative discussion are a useful new addition to the manuscript.

We thank the reviewer for these suggestions.

Moreover, they added new post- FL CFU flow cytometry data (SFig 7), which are described at lines 432-439. Here, the authors state that: “KO cells can commit to the erythroid lineage and generate erythroid progenitors, whereas progression to later stages of erythroid maturation is compromised…. the mean fluorescence intensity (MFI) of Ter119 expression was also reduced in KO cells, further supporting the conclusion that erythroid maturation is impaired”. Furthermore, in Discussion section lines 511-512 it is now stated that: “FACS analysis of CFU colonies showed that erythroid progenitors from KO FL are able to commit to the erythroid lineage and express CD71, although they fail to undergo further differentiation”.
I would suggest changing these statements and to be very careful with any conclusion about “impaired maturation” drawn from these data, because neither the % of Ter119+ cells or Ter119 MFI show any significant difference between genotypes (new S Fig7), but only slight trends with noticeable variabilility.

Overall, the revised manuscript has been strengthened, and I am satisfied with the authors’ response. Provided the authors amend the statements above, I now support its publication.

According to the reviewers’ suggestions, we have now modified the text at line 432-439 and 511-512, being more careful about our conclusions.
