# Supplementary material for: Impaired erythroid maturation in murine embryos upon loss of the preeclampsia-associated serine protease prostasin Prostasin loss impairs erythropoiesis
Source: PLoS One. 2026 Oct 16;21(10):e0360958. doi: 10.1371/journal.pone.0360958 (PMC13674711; doi:10.1371/journal.pone.0360958)
Supplement: Peer review history

# Peer review: Decision Letter 0

**DOI:** 10.1371/journal.pone.0360958.r001

**Authors:** Michael Schubert

19 Apr 2026

Dear Dr. Di Carlo,

Thank you for submitting your manuscript to PLOS ONE. After careful consideration, we feel that it has merit but does not fully meet PLOS ONE’s publication criteria as it currently stands. Therefore, we invite you to submit a revised version of the manuscript that comprehensively addresses the points raised during the review process.

Please submit your revised manuscript by Jun 03 2026 11:59PM. If you will need more time than this to complete your revisions, please reply to this message or contact the journal office at plosone@plos.org. When you're ready to submit your revision, log on to https://www.editorialmanager.com/pone/ and select the 'Submissions Needing Revision' folder to locate your manuscript file.

- A letter that responds to each point raised by the academic editor and reviewer(s). You should upload this letter as a separate file labeled 'Response to Reviewers'.
- A marked-up copy of your manuscript that highlights changes made to the original version. You should upload this as a separate file labeled 'Revised Manuscript with Track Changes'.
- An unmarked version of your revised paper without tracked changes. You should upload this as a separate file labeled 'Manuscript'.

If you would like to make changes to your financial disclosure, please include your updated statement in your cover letter. Guidelines for resubmitting your figure files are available below the reviewer comments at the end of this letter.

If applicable, we recommend that you deposit your laboratory protocols in protocols.io to enhance the reproducibility of your results. Protocols.io assigns your protocol its own identifier (DOI) so that it can be cited independently in the future. For instructions see: https://journals.plos.org/plosone/s/submission-guidelines#loc-laboratory-protocols. Additionally, PLOS ONE offers an option for publishing peer-reviewed Lab Protocol articles, which describe protocols hosted on protocols.io. Read more information on sharing protocols at https://plos.org/protocols?utm_medium=editorial-email&utm_source=authorletters&utm_campaign=protocols.

As the corresponding author, your ORCID iD is verified in the submission system and will appear in the published article. PLOS supports the use of ORCID, and we encourage all coauthors to register for an ORCID iD and use it as well. Please encourage your coauthors to verify their ORCID iD within the submission system before final acceptance, as unverified ORCID iDs will not appear in the published article. *Only*  the individual author can complete the verification step; PLOS staff *cannot*  verify ORCID iDs on behalf of authors.

We look forward to receiving your revised manuscript.

Kind regards,

Michael Schubert

Academic Editor

PLOS One

**Journal Requirements:**

When submitting your revision, we need you to address these additional requirements.

1. Please ensure that your manuscript meets PLOS ONE's style requirements, including those for file naming. The PLOS ONE style templates can be found at

https://journals.plos.org/plosone/s/file?id=wjVg/PLOSOne_formatting_sample_main_body.pdf and

https://journals.plos.org/plosone/s/file?id=ba62/PLOSOne_formatting_sample_title_authors_affiliations.pdf

2. Thank you for stating the following financial disclosure:

“This research has been funded by Swiss National Science Foundation (grant 31003A-182478/1 and 31003A-163347) to Edith Hummler, unrestricted UNIL funds to Olaia Naveiras.”

Please state what role the funders took in the study.  If the funders had no role, please state: "The funders had no role in study design, data collection and analysis, decision to publish, or preparation of the manuscript."

If this statement is not correct you must amend it as needed.

Please include this amended Role of Funder statement in your cover letter; we will change the online submission form on your behalf.

3. Please note that funding information should not appear in any section or other areas of your manuscript. We will only publish funding information present in the Funding Statement section of the online submission form. Please remove any funding-related text from the manuscript.

4. PLOS ONE now requires that authors provide the original uncropped and unadjusted images underlying all blot or gel results reported in a submission’s figures or Supporting Information files. This policy and the journal’s other requirements for blot/gel reporting and figure preparation are described in detail at https://journals.plos.org/plosone/s/figures#loc-blot-and-gel-reporting-requirements and https://journals.plos.org/plosone/s/figures#loc-preparing-figures-from-image-files. When you submit your revised manuscript, please ensure that your figures adhere fully to these guidelines and provide the original underlying images for all blot or gel data reported in your submission. See the following link for instructions on providing the original image data: https://journals.plos.org/plosone/s/figures#loc-original-images-for-blots-and-gels.

In your cover letter, please note whether your blot/gel image data are in Supporting Information or posted at a public data repository, provide the repository URL if relevant, and provide specific details as to which raw blot/gel images, if any, are not available. Email us at plosone@plos.org if you have any questions.

5. If the reviewer comments include a recommendation to cite specific previously published works, please review and evaluate these publications to determine whether they are relevant and should be cited. There is no requirement to cite these works unless the editor has indicated otherwise.

[Note: HTML markup is below. Please do not edit.]

Reviewers' comments:

Reviewer's Responses to Questions

**Comments to the Author**

1. Is the manuscript technically sound, and do the data support the conclusions?

Reviewer #1: Yes

Reviewer #2: Yes

**********

2. Has the statistical analysis been performed appropriately and rigorously?

Reviewer #1: Yes

Reviewer #2: I Don't Know

**********

3. Have the authors made all data underlying the findings in their manuscript fully available?

The PLOS Data policy

Reviewer #1: Yes

Reviewer #2: Yes

**********

4. Is the manuscript presented in an intelligible fashion and written in standard English?

Reviewer #1: Yes

Reviewer #2: Yes

**********

Reviewer #1: In this study, the authors identified defective erythroid maturation in Prss8-/- embryos at E11.5 and E12.5 in the yolk sac, placenta, and fetal liver. They propose that prostasin, encoded by the Prss8 gene, regulates primitive erythropoiesis, despite its absence in erythroid cells during this developmental window. Instead, they observed impaired vascular remodeling in the yolk sac of Prss8-/- embryos. Based on these findings, they suggest that prostasin plays a previously unrecognized role in developmental erythropoiesis and in vascular patterning in both the placenta and embryo.

This is a well executed, technically sound, and interesting study by a research group that has long been engaged in prostasin research. The data are convincing, and the study provides valuable insights. This reviewer has several comments that may further strengthen the manuscript.

1) In Fig. 1, the authors show distinct prostasin expression in the yolk sac tissue. However, it remains unclear which specific cell types express this protein in the yolk sac. Immunohistochemical analysis or in situ hybridization to identify prostasin expressing cells would greatly enhance the manuscript.

2) It is plausible that yolk sac endothelial cells, as well as endothelial cells in the placenta and liver sinusoids, express prostasin and thereby support developmental erythropoiesis. This reviewer strongly recommends examining prostasin expression in endothelial cells. If endothelial cells indeed express prostasin, co culture experiments using Prss8-deficient or wild-type endothelial cells with HSCs or erythroblasts would be highly informative.

3) Do erythroid cells express PAR2?

4) It would be interesting to know whether the observed phenotype depends on the protease activity of prostasin. Please discuss this point.

Reviewer #2: The study by Di Carlo et al. investigates the role of the membrane-bound serine protease prostasin (encoded by Prss8) during murine embryonic development. Prss8 deficiency leads to embryonic lethality around E14.5. The authors previously demonstrated that these embryos show impaired placental labyrinth maturation. They now demonstrate that Prss8 deficient embryos also show defective vascular remodeling in the yolk sac. A significant finding is the reduced frequency of both primitive and definitive erythroblasts in the circulation and hematopoietic tissues (yolk sac, placenta, and fetal liver) at E11.5 and E12.5. Single-cell RNA sequencing (scRNA-seq) of WT and Prss8 deficient AGM regions reveals that Prss8-/- primitive erythroid cells have a unique transcriptomic profile, including the downregulation of fetal globin genes and the upregulation of ribosomal protein genes. Because prostasin is not expressed in the erythroid cells themselves, the authors conclude it regulates erythropoiesis through a cell-extrinsic mechanism likely linked to the vascular niche. Overall, the study is well-conceived and provides valuable insights into the non-cell-autonomous role of prostasin in erythropoiesis; however, I have several concerns, which should be addressed before the manuscript is suitable for publication.

Major points

1. The authors suggest that prostasin regulates erythropoiesis in a cell-extrinsic manner because the protease is expressed in the vasculature but not in erythroid cells. However, it remains unclear whether the erythroid maturation defect is a direct consequence of missing prostasin signaling in the niche or a secondary effect of the generalized vascular and placental failure (e.g., hypoxia or nutrient deficiency). The manuscript would benefit from a more detailed discussion or additional experimental evidence (such as in vitro co-culture) to decouple these two phenotypes.

2. While a cell-extrinsic role is proposed, the manuscript does not identify the specific target(s) of prostasin in this context. Given that prostasin is known to activate the epithelial sodium channel (ENaC) and Protease-Activated Receptor 2 (PAR2) in other tissues, the authors should discuss whether these pathways are active in the embryonic vascular niche and if their dysregulation could explain the observed phenotype.

3. The authors conclude that embryonic lethality is "presumably due -at least in part- to anemia". However, the described defects in the placental labyrinth and yolk sac vascular remodeling are often independently sufficient to cause lethality. A more critical comparison of the severity of the anemia versus the placental failure would be needed to support the claim that anemia is a major driver of the lethality.

4. While the study includes an interesting analysis of globins in the AGM (Fig. 5), a parallel analysis in the yolk sac is missing. Since the YS appears to be the only tissue expressing Prss8, it is crucial to investigate the globin status in this district. If scRNA-seq data is unavailable for this tissue, the authors should perform bulk qPCR or analysis on sorted cell populations to confirm whether this phenotype is also present in the protein's primary site of expression.

5. In several key experiments (e.g., Fig. 1A and Fig. 3G, H), the number of biological replicates appears somewhat limited. While results are reported as non-significant, the data trends suggest that increasing the sample size might reveal statistically significant differences. If possible, the authors could increase the number of replicates for these experiments to avoid false negatives and strengthen their conclusions regarding Prss8 function.

6. The Materials and Methods section lacks details regarding the Western Blotting. It is necessary to specify the amount of protein loaded per well (µg), as well as full details for the antibodies used (catalog number, supplier, and dilution) to ensure reproducibility.

7. The data show the absence of Prss8 protein in the E9.5 embryo and the E11.5 fetal liver (FL) or AGM. However, no Western Blotting analysis of the placenta is included. Given the embryonic lethality linked to placental defects, placenta (or specifically labyrinth) data should be included.

8. The decrease in CFU in the AGM and FL shown in Fig. 7H and K is attributed to a decrease in erythroid colonies, although these have not been scored separately in the M3434 assay. Can the authors provide data showing the number of erythroid colonies separately here? Also in this paragraph (lines 389-399) the authors seem to confuse the “complete hematopoietic CFU progenitor assays” (Figs 7F and I), as these are actually shown in Fig. 7G-K. Vice versa for the erythroid-specific CFU assay, which is not shown in Figs 7H and K as indicated but in Fig. 7F-I.

Minor points

1. Lines 213-214: “Vascular quantification at E12.5 revealed a significantly reduced number of vessel branches and junctions and a lower maximum branch length (Fig 2C).” This statement is not supported by the data: the graph in 2C shows no difference in maximum branch length but instead shows a reduction in the junctions. Moreover, the authors claim that the defective vascular phenotype of the KO was not evident at E11.5 but Figure 2E does show smaller arteries in the KO. I would suggest performing the vascular quantification analysis at E11.5 (and possibly at E10.5 as well) as additional time points to confirm the timing of the onset of the vascular remodeling defects.

2. Lines 256-258: “H&E staining of the yolk sac at E12.5 revealed the presence of a vast majority of nucleated erythroblasts within vessels of WT embryos and significantly fewer nucleated erythroblasts in the KO (Fig 3A)” No statistical test was performed to evaluate statistical significance here, so this statement should be changed. Moreover, the images shown in Fig 2A appear to show two different regions of the YS. Arteries are visible in the WM image presented in Figure 2B so it should be possible to identify similar regions.

3. I would suggest showing individual channels in Fig. 3B for clarity.

4. The presence of cytoplasmic vacuoles in Prss8-/- erythroblasts is noted as a key morphological defect. The authors should comment on whether these vacuoles represent autophagic vesicles, degenerating organelles, or another form of cellular stress.

5. Page 3, lines 69-73: Both the erythrocytes derived by the EMP wave and the third wave are referred as “definitive”. This can be confusing and the wording in this section should be changed. Also, to my knowledge there is no conclusive evidence that bona fide HSCs actually emerge in the YS.

6. Page 14, line 377: reference 43 appears to be incorrect.

**********

what does this mean?). If published, this will include your full peer review and any attached files.

If you choose “no”, your identity will remain anonymous but your review may still be made public.

**Do you want your identity to be public for this peer review?**  For information about this choice, including consent withdrawal, please see our Privacy Policy

Reviewer #1: No

Reviewer #2: No

**********

[NOTE: If reviewer comments were submitted as an attachment file, they will be attached to this email and accessible via the submission site. Please log into your account, locate the manuscript record, and check for the action link "View Attachments". If this link does not appear, there are no attachment files.]

To ensure your figures meet our technical requirements, please review our figure guidelines: https://journals.plos.org/plosone/s/figures

You may also use PLOS’s free figure tool, NAAS, to help you prepare publication quality figures: https://journals.plos.org/plosone/s/figures#loc-tools-for-figure-preparation.

NAAS will assess whether your figures meet our technical requirements by comparing each figure against our figure specifications.

# Author comment: Author response to Decision Letter 1

**DOI:** 10.1371/journal.pone.0360958.r002

11 Aug 2026

Reviewer #1: In this study, the authors identified defective erythroid maturation in Prss8-/- embryos at E11.5 and E12.5 in the yolk sac, placenta, and fetal liver. They propose that prostasin, encoded by the Prss8 gene, regulates primitive erythropoiesis, despite its absence in erythroid cells during this developmental window. Instead, they observed impaired vascular remodeling in the yolk sac of Prss8-/- embryos. Based on these findings, they suggest that prostasin plays a previously unrecognized role in developmental erythropoiesis and in vascular patterning in both the placenta and embryo.

This is a well executed, technically sound, and interesting study by a research group that has long been engaged in prostasin research. The data are convincing, and the study provides valuable insights. This reviewer has several comments that may further strengthen the manuscript.

1) In Fig. 1, the authors show distinct prostasin expression in the yolk sac tissue. However, it remains unclear which specific cell types express this protein in the yolk sac. Immunohistochemical analysis or in situ hybridization to identify prostasin expressing cells would greatly enhance the manuscript.

Animal experimentation is tightly regulated and controlled by local and Swiss authorities. Due to the given timeline for the revision, we could not perform further yolk sac analysis, but we have now addressed this issue through sc-RNA data analyses. At both E10.50 and E11.5, we found Prss8 being mostly -if not exclusively- expressed within the extra-embryonic (EXE) endoderm cluster. This is now included in the manuscript as new SFig.1 and discussed in line 239 to 243.

2) It is plausible that yolk sac endothelial cells, as well as endothelial cells in the placenta and liver sinusoids, express prostasin and thereby support developmental erythropoiesis. This reviewer strongly recommends examining prostasin expression in endothelial cells. If endothelial cells indeed express prostasin, co culture experiments using Prss8-deficient or wild-type endothelial cells with HSCs or erythroblasts would be highly informative.

We thank the reviewer for this interesting point. Due to local and Swiss regulation for animal experimentation, and the given timeline for the revision, co-culture experiments were not feasible, and might be subject of a follow-up study. However, our scRNA-seq analysis of the yolk sac showed that endothelial cells do not express Prss8. This is now included in the manuscript as SFig.1.

3) Do erythroid cells express PAR2?

We now analyzed PAR2 gene expression (F2rl1) in the yolk sac using our scRNA-seq data and found that PAR2 is not expressed by erythroid cells, but mainly if not exclusively by the extraembryonic endoderm (EXE) cluster at E10.5 or E11.5, interestingly overlapping with Prss8 expression. This is now included in the manuscript in SFig1.

4) It would be interesting to know whether the observed phenotype depends on the protease activity of prostasin. Please discuss this point.

This is an interesting point; however, due to the small embryo size as well as the problem of specifically monitoring prostasin activity, this will be a difficult task. We now further discuss this possibility in the manuscript lines 491 to 502.

Reviewer #2: The study by Di Carlo et al. investigates the role of the membrane-bound serine protease prostasin (encoded by Prss8) during murine embryonic development. Prss8 deficiency leads to embryonic lethality around E14.5. The authors previously demonstrated that these embryos show impaired placental labyrinth maturation. They now demonstrate that Prss8 deficient embryos also show defective vascular remodeling in the yolk sac. A significant finding is the reduced frequency of both primitive and definitive erythroblasts in the circulation and hematopoietic tissues (yolk sac, placenta, and fetal liver) at E11.5 and E12.5. Single-cell RNA sequencing (scRNA-seq) of WT and Prss8 deficient AGM regions reveals that Prss8-/- primitive erythroid cells have a unique transcriptomic profile, including the downregulation of fetal globin genes and the upregulation of ribosomal protein genes. Because prostasin is not expressed in the erythroid cells themselves, the authors conclude it regulates erythropoiesis through a cell-extrinsic mechanism likely linked to the vascular niche. Overall, the study is well-conceived and provides valuable insights into the non-cell-autonomous role of prostasin in erythropoiesis; however, I have several concerns, which should be addressed before the manuscript is suitable for publication.

Major points

1. The authors suggest that prostasin regulates erythropoiesis in a cell-extrinsic manner because the protease is expressed in the vasculature but not in erythroid cells. However, it remains unclear whether the erythroid maturation defect is a direct consequence of missing prostasin signaling in the niche or a secondary effect of the generalized vascular and placental failure (e.g., hypoxia or nutrient deficiency). The manuscript would benefit from a more detailed discussion or additional experimental evidence (such as in vitro co-culture) to decouple these two phenotypes.

We agree that it would be interesting to follow this in more detail. Due to Swiss and local regulation for animal experimentation, and the given timeline for the revision, such experiments are now not feasible and should certainly be the subject of a follow-up study. We now further discuss these possibilities in the manuscript (lines 485 to 491).

2. While a cell-extrinsic role is proposed, the manuscript does not identify the specific target(s) of prostasin in this context. Given that prostasin is known to activate the epithelial sodium channel (ENaC) and Protease-Activated Receptor 2 (PAR2) in other tissues, the authors should discuss whether these pathways are active in the embryonic vascular niche and if their dysregulation could explain the observed phenotype.

As mentioned above as comment to Reviewer 1, we have now analyzed PAR2 gene expression in the yolk sac through scRNA-seq data and have found that F2rl1 (encoding for PAR2) is expressed mainly if not exclusively by the extraembryonic endoderm (EXE) cluster, interestingly overlapping with Prss8 expression. ENaC (alpha subunit) Scnn1g is not expressed in the yolk sac, therefore suggesting that the Prss8-ENaC axis may not play a role in Prss8-driven embryonic lethality. This is now included in SFig1 and further discussed in the manuscript (lines 491 to 502).

3. The authors conclude that embryonic lethality is "presumably due -at least in part- to anemia". However, the described defects in the placental labyrinth and yolk sac vascular remodeling are often independently sufficient to cause lethality. A more critical comparison of the severity of the anemia versus the placental failure would be needed to support the claim that anemia is a major driver of the lethality.

We agree with the reviewer and have modified the text accordingly.

4. While the study includes an interesting analysis of globins in the AGM (Fig. 5), a parallel analysis in the yolk sac is missing. Since the YS appears to be the only tissue expressing Prss8, it is crucial to investigate the globin status in this district. If scRNA-seq data is unavailable for this tissue, the authors should perform bulk qPCR or analysis on sorted cell populations to confirm whether this phenotype is also present in the protein's primary site of expression.

We newly analyzed the expression of globin genes through scRNA.seq analyses of WT and KO YS at E10.5 and E11.5, and found no decline for any fetal globin genes across erythroid clusters in the KO (SFig1 C-D). In comparison, those genes were strongly reduced in the KO AGM at E11.5 (Fig 5G). This suggests that Prrs8 influences erythroblast migration and/or further differentiation rather than the generation of primitive erythroid progenitors in the yolk sac. This is now included in SFig.1 and further discussed in the manuscript lines 521 to 524.

5. In several key experiments (e.g., Fig. 1A and Fig. 3G, H), the number of biological replicates appears somewhat limited. While results are reported as non-significant, the data trends suggest that increasing the sample size might reveal statistically significant differences. If possible, the authors could increase the number of replicates for these experiments to avoid false negatives and strengthen their conclusions regarding Prss8 function.

Due to local and Swiss regulation on animal experimentation, and the given timeline for the revision, repetition of such experiments is now not feasible. For Fig. 1A, we have now reformulated the conclusion more carefully. In Fig.3G,H, there is a suggested increased number of colonies in KO YS-derived cultures, and this might likely be due to the difference between progenitor activity in vitro and in vivo. It is plausible that erythroid progenitors accumulate and that terminal differentiation into erythrocytes is defective in the KO. This is now mentioned in the manuscript.

6. The Materials and Methods section lacks details regarding the Western Blotting. It is necessary to specify the amount of protein loaded per well (µg), as well as full details for the antibodies used (catalog number, supplier, and dilution) to ensure reproducibility.

We apologize for any missing information and have now included all the details in a paragraph of “Material and methods” section.

7. The data show the absence of Prss8 protein in the E9.5 embryo and the E11.5 fetal liver (FL) or AGM. However, no Western Blotting analysis of the placenta is included. Given the embryonic lethality linked to placental defects, placenta (or specifically labyrinth) data should be included.

We previously analyzed the mRNA transcription profile of murine Prss8 in the placenta of E11.5 – E16.5 embryos (PMID: 23405214) and found a peak of expression in E13.5 WT embryos. This increase is missing in Prss8 KO embryos and thus precedes the failure of placental maturation underlining a time-dependent requirement of Prss8 expression during mouse embryogenesis. In our study, we focused on earlier embryonic time-points (E9.5 – E11.5) that revealed an impaired erythroid maturation in the AGM and fetal liver in Prss8 KO embryos before the placental failure becomes evident. Currently, we cannot exclude the contribution of placenta failure to this phenotype, but this will certainly be subject of a follow-up study. The issue is now discussed in lines 486-491 of the manuscript.

8. The decrease in CFU in the AGM and FL shown in Fig. 7H and K is attributed to a decrease in erythroid colonies, although these have not been scored separately in the M3434 assay. Can the authors provide data showing the number of erythroid colonies separately here? Also in this paragraph (lines 389-399) the authors seem to confuse the “complete hematopoietic CFU progenitor assays” (Figs 7F and I), as these are actually shown in Fig. 7G-K. Vice versa for the erythroid-specific CFU assay, which is not shown in Figs 7H and K as indicated but in Fig. 7F-I.

We thank the reviewer for this point. Since the StemCell Vision linked method does not allow for the reliable separation of erythroid colonies in the M3434 medium conditions, we have performed FACS analyses of FL derived cells after 7 days of culture to evaluate erythroid lineage differentiation on a single cell level after CFU assays. The new data is now presented as new SFig.7 and discussed in lines 432 to 440 of the manuscript. We have also corrected the order of panels on Fig. 7.

Minor points

1. Lines 213-214: “Vascular quantification at E12.5 revealed a significantly reduced number of vessel branches and junctions and a lower maximum branch length (Fig 2C).” This statement is not supported by the data: the graph in 2C shows no difference in maximum branch length but instead shows a reduction in the junctions. Moreover, the authors claim that the defective vascular phenotype of the KO was not evident at E11.5 but Figure 2E does show smaller arteries in the KO. I would suggest performing the vascular quantification analysis at E11.5 (and possibly at E10.5 as well) as additional time points to confirm the timing of the onset of the vascular remodeling defects.

We thank the reviewer for raising this point. We have now corrected the sentence accordingly. We conclude that the vascular defect became evident at E11.5 based on multiple complementary analyses, namely gross morphology (Fig.2A), YS immune fluorescence (IF) (Fig.2B), and bulk RNA sequencing DEG analysis between E10.5 and E11.5 (Fig.2I), all of which consistently support a vascular defect at E11.5. Therefore, we concluded that additional analyses of E10.5 YS IF would not further strengthen this point.

2. Lines 256-258: “H&E staining of the yolk sac at E12.5 revealed the presence of a vast majority of nucleated erythroblasts within vessels of WT embryos and significantly fewer nucleated erythroblasts in the KO (Fig 3A)” No statistical test was performed to evaluate statistical significance here, so this statement should be changed. Moreover, the images shown in Fig 2A appear to show two different regions of the YS. Arteries are visible in the WM image presented in Figure 2B so it should be possible to identify similar regions.

We have changed the text accordingly.

3. I would suggest showing individual channels in Fig. 3B for clarity.

We have now modified Fig.3B accordingly and included images for each channel.

4. The presence of cytoplasmic vacuoles in Prss8-/- erythroblasts is noted as a key morphological defect. The authors should comment on whether these vacuoles represent autophagic vesicles, degenerating organelles, or another form of cellular stress.

We thank the reviewer to raise this point. We have now discussed this in the manuscript lines 537 to 549.

5. Page 3, lines 69-73: Both the erythrocytes derived by the EMP wave and the third wave are referred as “definitive”. This can be confusing and the wording in this section should be changed. Also, to my knowledge there is no conclusive evidence that bona fide HSCs actually emerge in the YS.

We have now changed the text accordingly and refer to EMP-derived erythrocytes and HSC-definitive erythrocytes for better clarity.

6. Page 14, line 377: reference 43 appears to be incorrect.

We thank the reviewer for noticing this mistake. We now refer to the correct reference.

Attachment Submitted filename: Response to Reviewers.docx

# Peer review: Decision Letter 1

**DOI:** 10.1371/journal.pone.0360958.r003

**Authors:** Michael Schubert

9 Sep 2026

Dear Dr. Di Carlo,

Thank you for submitting your manuscript to PLOS One. After careful consideration, we feel that it has merit but does not fully meet PLOS One’s publication criteria as it currently stands. Therefore, we invite you to submit a revised version of the manuscript that addresses the points raised during the review process.

Please submit your revised manuscript by Oct 24 2026 11:59PM. If you will need more time than this to complete your revisions, please reply to this message or contact the journal office at plosone@plos.org. When you're ready to submit your revision, log on to https://www.editorialmanager.com/pone/ and select the 'Submissions Needing Revision' folder to locate your manuscript file.

- A letter that responds to each point raised by the academic editor and reviewer(s). You should upload this letter as a separate file labeled 'Response to Reviewers'.
- A marked-up copy of your manuscript that highlights changes made to the original version. You should upload this as a separate file labeled 'Revised Manuscript with Track Changes'.
- An unmarked version of your revised paper without tracked changes. You should upload this as a separate file labeled 'Manuscript'.

If you would like to make changes to your financial disclosure, please include your updated statement in your cover letter. Guidelines for resubmitting your figure files are available below the reviewer comments at the end of this letter.

If applicable, we recommend that you deposit your laboratory protocols in protocols.io to enhance the reproducibility of your results. Protocols.io assigns your protocol its own identifier (DOI) so that it can be cited independently in the future. For instructions see: https://journals.plos.org/plosone/s/submission-guidelines#loc-laboratory-protocols. Additionally, PLOS One offers an option for publishing peer-reviewed Lab Protocol articles, which describe protocols hosted on protocols.io. Read more information on sharing protocols at https://plos.org/protocols?utm_medium=editorial-email&utm_source=authorletters&utm_campaign=protocols.

As the corresponding author, your ORCID iD is verified in the submission system and will appear in the published article. PLOS supports the use of ORCID, and we encourage all coauthors to register for an ORCID iD and use it as well. Please encourage your coauthors to verify their ORCID iD within the submission system before final acceptance, as unverified ORCID iDs will not appear in the published article. *Only*  the individual author can complete the verification step; PLOS staff *cannot*  verify ORCID iDs on behalf of authors.

We look forward to receiving your revised manuscript.

Kind regards,

Michael Schubert

Academic Editor

PLOS One

Journal Requirements:

1. If the reviewer comments include a recommendation to cite specific previously published works, please review and evaluate these publications to determine whether they are relevant and should be cited. There is no requirement to cite these works unless the editor has indicated otherwise.

2. Please review your reference list to ensure that it is complete and correct. If you have cited papers that have been retracted, please include the rationale for doing so in the manuscript text, or remove these references and replace them with relevant current references. Any changes to the reference list should be mentioned in the rebuttal letter that accompanies your revised manuscript. If you need to cite a retracted article, indicate the article’s retracted status in the References list and also include a citation and full reference for the retraction notice.

[Note: HTML markup is below. Please do not edit.]

Reviewer's Responses to Questions

**Comments to the Author**

Reviewer #1: All comments have been addressed

Reviewer #2: (No Response)

**********

2. Is the manuscript technically sound, and do the data support the conclusions?

Reviewer #1: Yes

Reviewer #2: Yes

**********

3. Has the statistical analysis been performed appropriately and rigorously?

Reviewer #1: Yes

Reviewer #2: Yes

**********

4. Have the authors made all data underlying the findings in their manuscript fully available?

The PLOS Data policy

Reviewer #1: Yes

Reviewer #2: Yes

**********

5. Is the manuscript presented in an intelligible fashion and written in standard English?

Reviewer #1: Yes

Reviewer #2: Yes

**********

Reviewer #1: The manuscript has been partially improved in response to my comments. Although a few issues remain to be addressed, I accept the authors’ rebuttal and have no further comments. Thank you for your responses.

Reviewer #2: In the revised manuscript, the authors have addressed my concerns by adding new analyses, amending claims and figures and expanding the discussion.

Specifically, they have now incorporated new scRNAseq data analysis of the YS (SFig 1), which shows that Prss8 is expressed in the extra-embryonic endoderm (EXE), though they see no decrease in fetal globins in the YS. Also, PAR2 appears to be expressed by the EXE. In the absence of any new functional data, these transcriptomic data and relative discussion are a useful new addition to the manuscript.

Moreover, they added new post- FL CFU flow cytometry data (SFig 7), which are described at lines 432-439. Here, the authors state that: “KO cells can commit to the erythroid lineage and generate erythroid progenitors, whereas progression to later stages of erythroid maturation is compromised…. the mean fluorescence intensity (MFI) of Ter119 expression was also reduced in KO cells, further supporting the conclusion that erythroid maturation is impaired”. Furthermore, in Discussion section lines 511-512 it is now stated that: “FACS analysis of CFU colonies showed that erythroid progenitors from KO FL are able to commit to the erythroid lineage and express CD71, although they fail to undergo further differentiation”.

I would suggest changing these statements and to be very careful with any conclusion about “impaired maturation” drawn from these data, because neither the % of Ter119+ cells or Ter119 MFI show any significant difference between genotypes (new S Fig7), but only slight trends with noticeable variabilility.

Overall, the revised manuscript has been strengthened, and I am satisfied with the authors’ response. Provided the authors amend the statements above, I now support its publication.

**********

what does this mean?). If published, this will include your full peer review and any attached files.

If you choose “no”, your identity will remain anonymous but your review may still be made public.

**Do you want your identity to be public for this peer review?**  For information about this choice, including consent withdrawal, please see our Privacy Policy

Reviewer #1: No

Reviewer #2: No

**********

[NOTE: If reviewer comments were submitted as an attachment file, they will be attached to this email and accessible via the submission site. Please log into your account, locate the manuscript record, and check for the action link "View Attachments". If this link does not appear, there are no attachment files.]

To ensure your figures meet our technical requirements, please review our figure guidelines: https://journals.plos.org/plosone/s/figures

You may also use PLOS’s free figure tool, NAAS, to help you prepare publication quality figures: https://journals.plos.org/plosone/s/figures#loc-tools-for-figure-preparation.

NAAS will assess whether your figures meet our technical requirements by comparing each figure against our figure specifications.

# Author comment: Author response to Decision Letter 2

**DOI:** 10.1371/journal.pone.0360958.r004

29 Sep 2026

Response to Reviewers

We would like to thank the reviewers for their constructive inputs and for giving us the chance to improve our manuscript. The text in results and discussion was amended as suggested.

Review Comments to the Author

Reviewer #1: The manuscript has been partially improved in response to my comments. Although a few issues remain to be addressed, I accept the authors’ rebuttal and have no further comments. Thank you for your responses.

We thank the reviewer that helped us improving our manuscript.

Reviewer #2: In the revised manuscript, the authors have addressed my concerns by adding new analyses, amending claims and figures and expanding the discussion.

Specifically, they have now incorporated new scRNAseq data analysis of the YS (SFig 1), which shows that Prss8 is expressed in the extra-embryonic endoderm (EXE), though they see no decrease in fetal globins in the YS. Also, PAR2 appears to be expressed by the EXE. In the absence of any new functional data, these transcriptomic data and relative discussion are a useful new addition to the manuscript.

We thank the reviewer for these suggestions.

Moreover, they added new post- FL CFU flow cytometry data (SFig 7), which are described at lines 432-439. Here, the authors state that: “KO cells can commit to the erythroid lineage and generate erythroid progenitors, whereas progression to later stages of erythroid maturation is compromised…. the mean fluorescence intensity (MFI) of Ter119 expression was also reduced in KO cells, further supporting the conclusion that erythroid maturation is impaired”. Furthermore, in Discussion section lines 511-512 it is now stated that: “FACS analysis of CFU colonies showed that erythroid progenitors from KO FL are able to commit to the erythroid lineage and express CD71, although they fail to undergo further differentiation”.

I would suggest changing these statements and to be very careful with any conclusion about “impaired maturation” drawn from these data, because neither the % of Ter119+ cells or Ter119 MFI show any significant difference between genotypes (new S Fig7), but only slight trends with noticeable variabilility.

Overall, the revised manuscript has been strengthened, and I am satisfied with the authors’ response. Provided the authors amend the statements above, I now support its publication.

According to the reviewers’ suggestions, we have now modified the text at line 432-439 and 511-512, being more careful about our conclusions.

Attachment Submitted filename: Response_to_Reviewers_auresp_2.docx

# Editor report: Decision Letter 2

**DOI:** 10.1371/journal.pone.0360958.r005

**Authors:** Michael Schubert

5 Oct 2026

Impaired erythroid maturation in murine embryos upon loss of the preeclampsia-associated serine protease prostasin

PONE-D-26-08825R2

Dear Dr. Di Carlo,

We’re pleased to inform you that your manuscript has been judged scientifically suitable for publication and will be formally accepted for publication once it meets all outstanding technical requirements.

Within one week, you’ll receive an e-mail detailing the required amendments. When these have been addressed, you’ll receive a formal acceptance letter and your manuscript will be scheduled for publication.

An invoice will be generated when your article is formally accepted. Please note, if your institution has a publishing partnership with PLOS and your article meets the relevant criteria, all or part of your publication costs will be covered. Please make sure your user information is up-to-date by logging into Editorial Manager at Editorial Manager® and clicking the ‘Update My Information' link at the top of the page. For questions related to billing, please contact billing support.

If your institution or institutions have a press office, please notify them about your upcoming paper to help maximize its impact. If they’ll be preparing press materials, please inform our press team as soon as possible -- no later than 48 hours after receiving the formal acceptance. Your manuscript will remain under strict press embargo until 2 pm Eastern Time on the date of publication. For more information, please contact onepress@plos.org.

Kind regards,

Michael Schubert

Academic Editor

PLOS One

# Editor report: Acceptance letter

**DOI:** 10.1371/journal.pone.0360958.r006

**Authors:** Michael Schubert

PONE-D-26-08825R2

PLOS One

Dear Dr. Di Carlo,

I'm pleased to inform you that your manuscript has been deemed suitable for publication in PLOS One. Congratulations! Your manuscript is now being handed over to our production team.

At this stage, our production department will prepare your paper for publication. This includes ensuring the following:

* All references, tables, and figures are properly cited

* All relevant supporting information is included in the manuscript submission,

* There are no issues that prevent the paper from being properly typeset

You will receive further instructions from the production team, including instructions on how to review your proof when it is ready. Please keep in mind that we are working through a large volume of accepted articles, so please give us a few days to review your paper and let you know the next and final steps.

Lastly, if your institution or institutions have a press office, please let them know about your upcoming paper now to help maximize its impact. If they'll be preparing press materials, please inform our press team within the next 48 hours. Your manuscript will remain under strict press embargo until 2 pm Eastern Time on the date of publication. For more information, please contact onepress@plos.org.

You will receive an invoice from PLOS for your publication fee after your manuscript has reached the completed accept phase. If you receive an email requesting payment before acceptance or for any other service, this may be a phishing scheme. Learn how to identify phishing emails and protect your accounts at https://explore.plos.org/phishing.

If we can help with anything else, please email us at customercare@plos.org.

Thank you for submitting your work to PLOS One and supporting open access.

Kind regards,

PLOS One Editorial Office Staff

on behalf of

Dr. Michael Schubert

Academic Editor

PLOS One
